# Boronic Acid Appended Naphthyl-Pyridinium Receptors as Chemosensors for Sugars

**DOI:** 10.1038/s41598-019-42812-8

**Published:** 2019-04-30

**Authors:** Angel Resendez, Sanjay V. Malhotra

**Affiliations:** 1 Department of Radiation Oncology, Stanford University School of Medicine, Stanford, California 94305 USA; 2 Radiology, Stanford University School of Medicine, Stanford, California 94305 USA

**Keywords:** Biomarkers, Fluorescent probes

## Abstract

There remains a need in clinics and research to have simple and sensitive detection systems that allow the detection and quantification of sugar markers of biomedical relevance such as sugars lactulose and mannitol for noninvasive gut permeability assessment. We have prepared a new class of boronic acid-appended naphthyl-pyridinium receptor compounds as chemosensors. These were studied for their ability to act as modular internal charge transfer (ICT) fluorescent probes or donor/acceptor pair ensembles where the receptor compound can act as a quencher for an anionic dye. As an ICT sensor, fluorescence intensity increased upon diol recognition, which stems from the neutralization of the pyridinium nitrogen that is perturbing the chromophoric properties. We found these ICT probes provide good sensitivity for disaccharide lactulose with low micromolar detection and quantification limits. In addition, their ability to form a non-fluorescent ground state complex with anionic reporter dyes, such as HPTS or TSPP, was examined as probes for various sugars. We have identified three receptor/quencher compounds with high quenching efficiency for anionic dyes. Subsequently, a range of sugars and sugar derivatives were tested for chemosenstivity of our probes. This study illustrates an approach for designing boronic acid-based chemoreceptors for the recognition and quantification of sugars and sugar derivatives.

## Introduction

The molecular recognition of carbohydrates and diol-containing compounds by boronic acid systems is a growing area of research interest, and such fluorescent probes based on boronic acid recognition for sugars and sugar derivatives have been explored^1^. Most notable and popular probes are classified as “one component systems” that utilize an internal charge transfer mechanism for signal generation^2,3^. Various fluorophores that are amenable to boronic acid tethering, e.g., anthracene^4^, naphthalene^5^, naphthalimide^6,7^, indol^8^, quinolinium^9,10^, benzo-thiophene^11^, and fluorene^12^, containing boronic acid tethered dyes have been reported to measure sugars and sugar derivatives in aqueous media. Similarly, boronic acid containing fluorescent receptors have been developed for sugar alcohols^13^, sugar acids^14–16^, and recently, sugar markers such as lactulose and mannitol^17^ for clinical applications^18,19^. Due to the structural nature of these sugars, boronic acids can adopt different conformations when binding to mannitol (sugar alcohol) as compared with cyclic aldoses (glucose) or ketoses (fructose)^20–22^. Consequently, designing selective boronic acid receptors for these sugar alcohols and lactulose poses a difficult challenge^23–25^. Because lactulose and mannitol are important sugar markers for gut permeability assessment, designing sensitive and selective sensors for sugar alcohols has attracted considerable attention in the field of sensor design. There is an increasing need for a simple and easy to use method for analyzing the sugar gut permeability markers that offers good sensitivity and alleviates wavelength interference from urine components (i.e. riboflavin, urobilin, urobilinogen etc.) during sample analysis.

**Figure 1:**
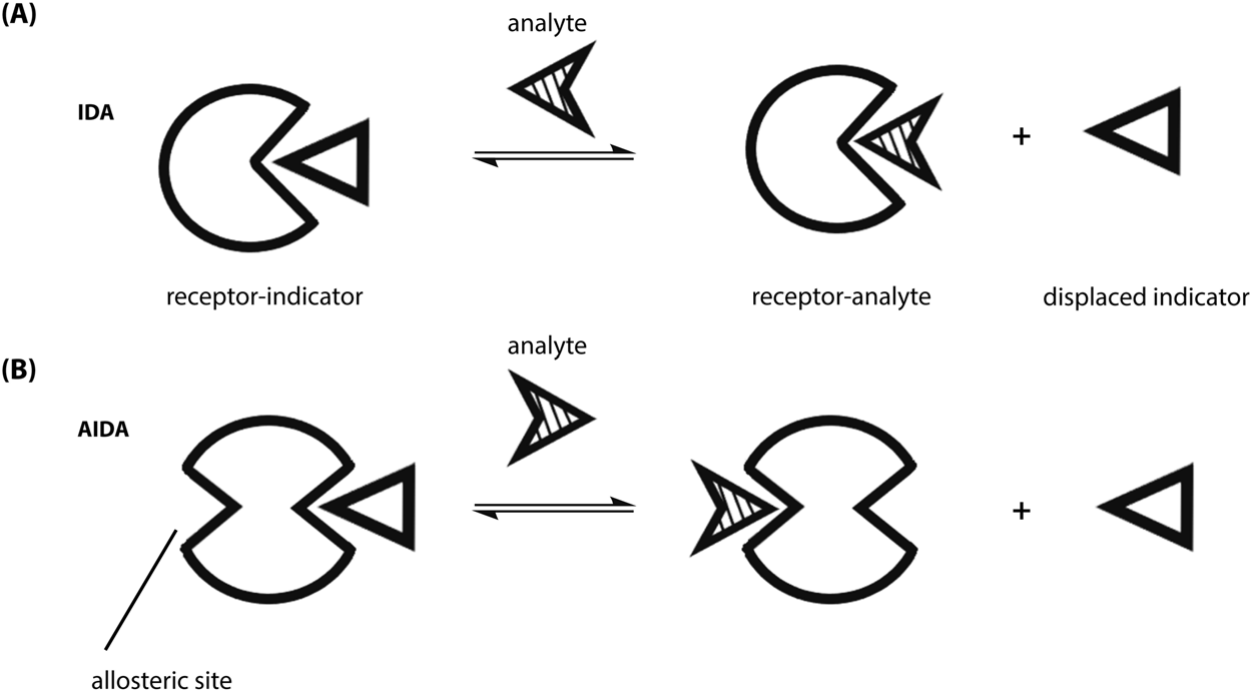
(**A**) Illustration of indicator displacement assay where the analyte competes for the receptor-indicator interaction. (**B**) Allosteric indicator displacement assay where the analyte binds at an allosteric site.

Indicator displacement assays (IDA) have been reported as competitive binding assay systems^26–32^, where the receptor and indicator (or reporter) units are two discrete entities which can provide advantages, such as signal modulation and ease of modification of receptor system over direct sensing (*i.e*., one-component systems) (Fig. 1). Singaram *et al*. demonstrated a two-component system and designed a series of boronic acid appended bipyridinium compounds that utilized an anionic dye (reporter) and a boronic acid-appended viologen (receptor) to develop a continuous glucose sensor^33^.

Two-component systems can be incorporated into a standard IDA, where an analyte displaces the indicator via allosteric interaction and the analyte cannot compete at the same binding site as the indicator. Instead, the analyte binds at another site, inducing a decrease in the affinity of the indicator for the receptor. This type of system refers to as an allosteric indicator displacement assay (AIDA). The two-component system relies on the cationic nature of the receptor compound to form a non-fluorescent ground state complex with the anionic fluorescent dye, 8-hydroxypyrene, 1,3,6-trisulfonic acid trisodium salt (HPTS), and a boronic acid–appended viologen (BBV) that acts as both a quencher and receptor. In the absence of sugar, a ground state complex forms by the coulombic attraction between the anionic dye and cationic quencher with a decrease of fluorescence intensity (as compared with free HPTS). When a sugar binds, the boronic acids convert to tetrahedral anionic boronate ester, which neutralizes the cationic viologen, diminishing its quenching efficiency and liberating HPTS. The fluorescent signal generated upon dissociation of the ground state complex is directly proportional to sugar concentration. Previous work demonstrated that the 4,4′-*o*-BBV bidentate receptor exhibited increased binding affinity due to the ortho substitution of the boronic acid motif^34^. The boronic acid sits close to the quaternary nitrogen, thus, able to participate in a favorable electrostatic interaction. Studies of several boronic acid-appended viologens that possess both bipyridinium or phenanthrolinium cores have been previously reported^35^. The ability of these viologens to quench the fluorescence of various anionic reporters is directly proportional to the number of cationic charges on the quencher^35–37^. To alleviate the need for a large number of cationic groups, we investigated new boronic acid-appended quenchers with ortho substitution for improved ability to (1) quench the fluorescence of an anionic reporter and (2) improve the sensitivity for the recognition of sugars and sugar derivatives. Herein, we report new boronic acid-appended naphthyl-pyridinium receptor compounds as discrete two-component fluorophore-receptor probes and their ability to quench the fluorescence of anionic dyes. The combination of each probe (receptor-dye) was investigated for their performance in recognizing various sugars and sugar derivatives.

## Results

**Figure 2:**
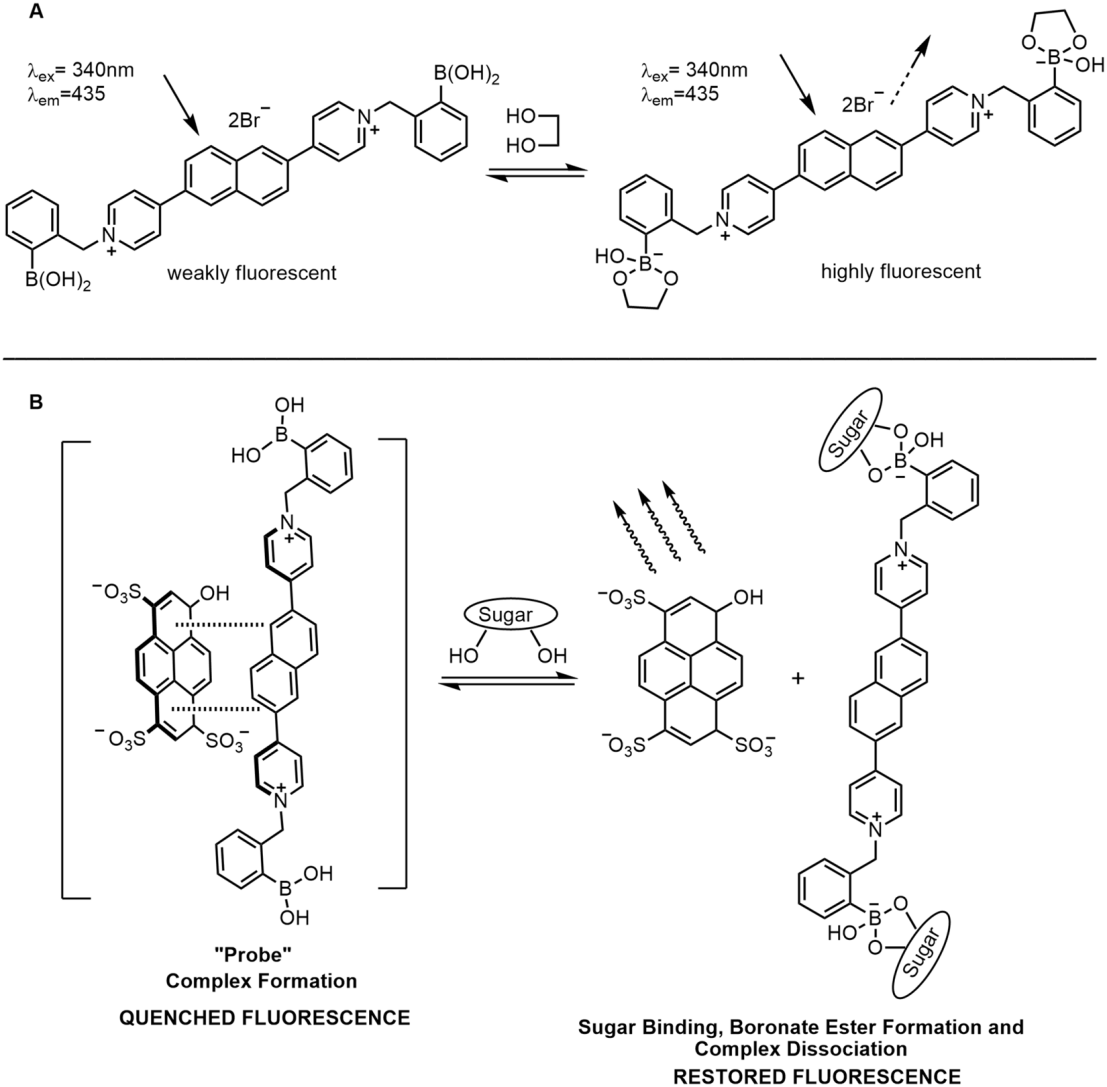
(**A**) Proposed formation of a fluorescent species upon diol recognition. (**B**) Proposed mechanism of signal transduction for a two-component fluorescent probe based on boronic acid–appended naphthyl-pyridinium compounds.

An indicator displacement “two-component” system can be used to detect sugars and sugar derivatives, as it provides flexibility in signal modulation and is less sensitive to pH changes. The flexibility of the two-component system is that it allows for tailoring of the quencher/receptor compound’s properties without affecting the photophysical properties of the reporter dye. This can improve the sensitivity, and also the selectivity of measuring sugar and sugar derivatives in complex media, such as urine or blood. With the ability to overcome sensitivity and/or selectivity challenges that come with boronic acid-based recognition systems, we pursued a naphthalene moiety as a new quencher along with anionic dyes such as HPTS and tetrakis (4-sulfophenyl) porphine (TSPP). Since naphthalene has its intrinsic fluorescence properties^38^, we first investigated these naphthyl-pyridinium receptors as potential new one-component systems to act as chemosensors for sugars and sugar derivatives. The previous study reported a naphthalene-based sensor for fructose recognition which utilizes the internal charge transfer (ICT) process for the generation of a fluorescent signal^5^. This sensor relies on the sp^2^-hybridized boron atom directly attached to the naphthalene chromophore, which can form a conjugated system and act as an electron acceptor. We envisioned designing a new type of boronic acid-based chemosensor that relies on quaternary nitrogen that is separate from the fluorophore unit, and primarily responsible for the ICT mechanism. To design this, we incorporated a pyridinium tethered to the fluorophore moiety acting as the electron acceptor to facilitate the excited state charge transfer. Upon converting the boron atom from sp^2^- to sp^3^-hybridization, it switches off the ICT process resulting in an increase of fluorescence with increasing sugar concentration. We hypothesized that a quarternized nitrogen could serve as an excellent electron acceptor for the excited singlet electron and this would alter the emissive properties of naphthalene upon the charge neutralization event from sp^2^- to sp^3^-hybridization change, thereby further inducing spectroscopic changes (Fig. 2A). Additionally, because of its conjugated π-system and cationic character, we hypothesized that these naphthyl-pyridinium receptor compounds would be excellent quenchers for the anionic dye HPTS or TSPP. Upon boronic acid-diol recognition of sugar, it would form boronate, thus neutralizing the quarternized nitrogen and liberating the anionic fluorophore for detection (Fig. 2B).

**Figure 3:**
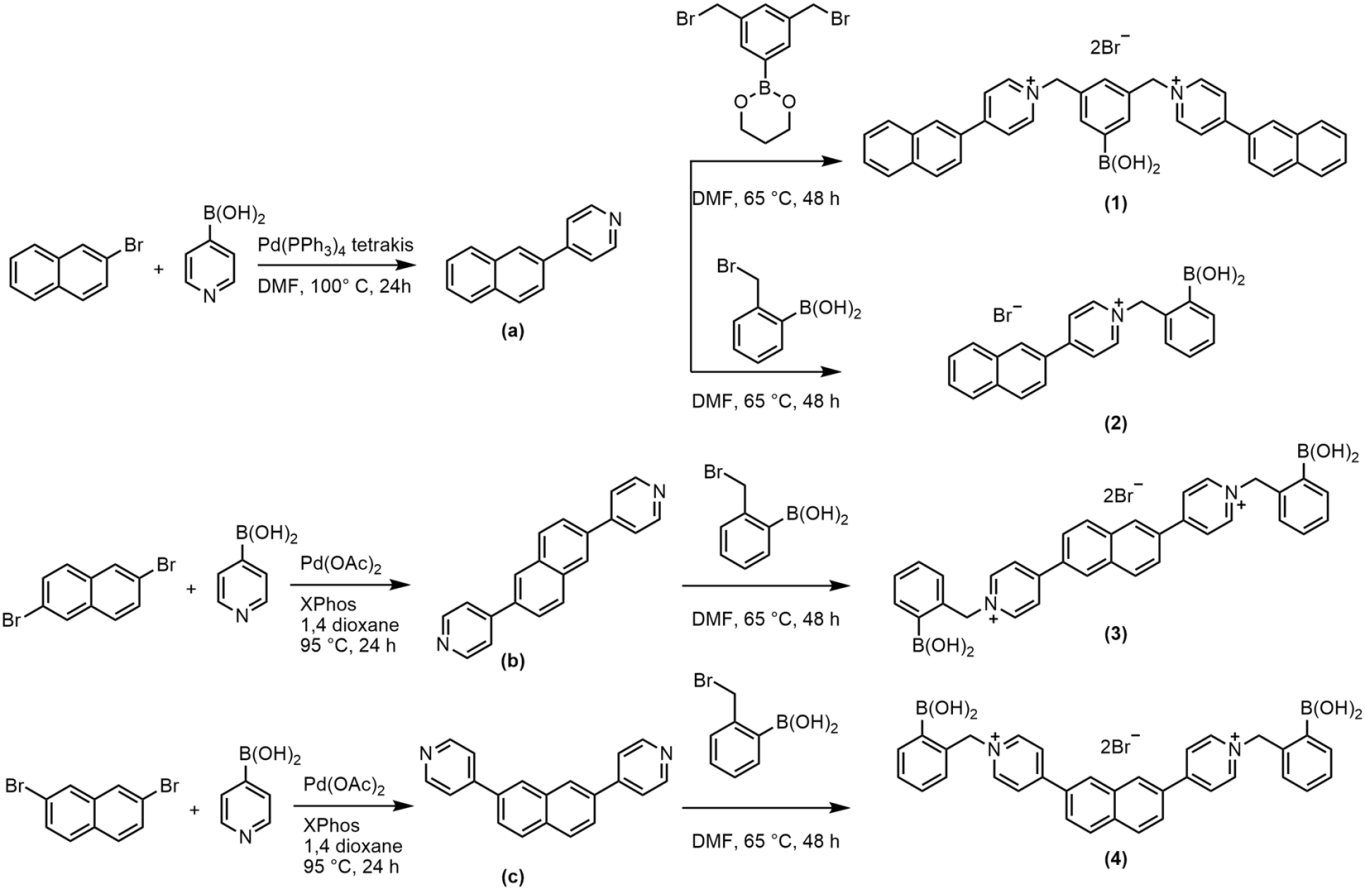
Synthetic route for obtaining the newly designed boronic acid appended naphthyl-pyridinium receptor compounds (**1–4**).

Starting with commercially available 1-bromo- or dibromo-naphthalene, each coupled precursor was obtained via Suzuki-Miyaura coupling, followed by nucleophilic substitution to afford the boronic acid appended naphthyl-pyridinium receptor salt compound (Fig. 3). The linking of naphthalene and pyridine aromatic systems provides a unique environment for complex formation with its anionic partner that would enhance the electron transfer, improving the ability to quench the fluorophore with minimal quencher concentration. Furthermore, this unique structural feature enables the receptor compound to undergo an ICT process, acting as a one-component system and facilitating participation in dual probe systems.

### Absorption and fluorescence studies of boronic acid appended naphthyl-pyridinium receptor compounds

**Figure 4:**
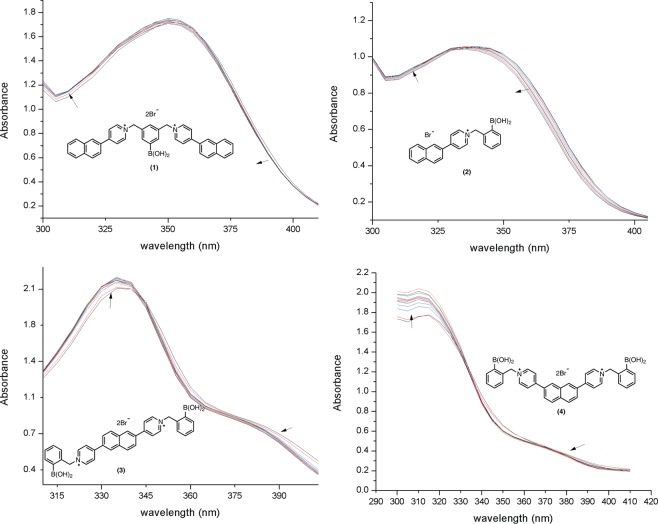
Absorption spectra of each naphthyl-pyridinium receptor compounds in 4% DMSO, 0.1 M sodium phosphate buffer pH 7.4 with increasing lactulose (0–40 mM in 4 mM increments).

**Figure 5:**
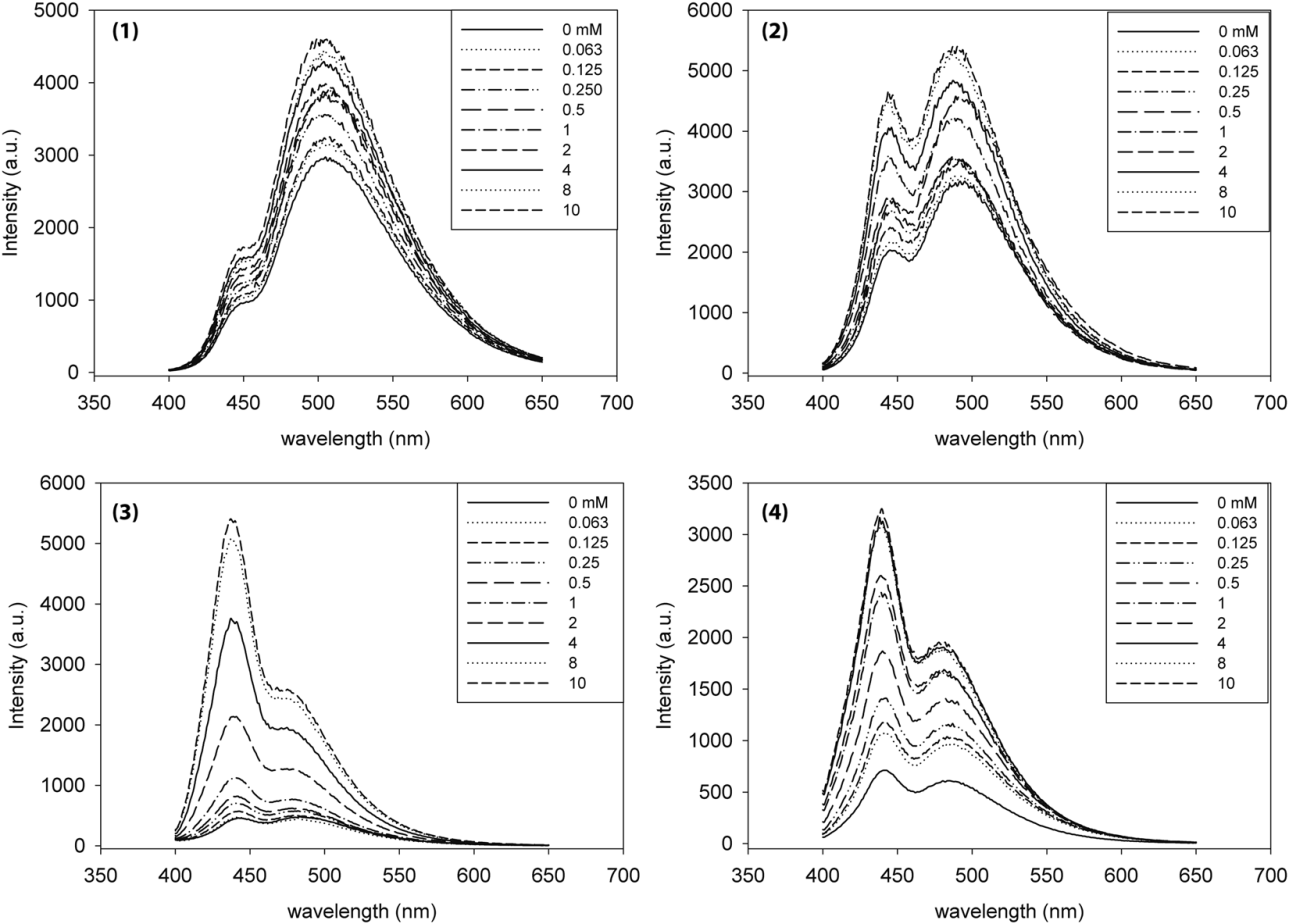
Fluorescence emission spectra each naphthyl-pyridinium receptor compounds (500 μM, **1** & **2**, 250 μM for **3** & **4**) with increasing lactulose 0–10 mM in 0.1 M sodium phosphate buffer pH 7.4. Excitation wavelengths used λ_ex_=300 for (**1**) and (**2**), and 340 nm for (**3**) and (**4**) receptor compound.

With naphthalene as the core moiety of each receptor compound, we anticipated these compounds would undergo photophysical changes upon recognition of a sugar. Therefore, the compound was examined for its absorbance changes to demonstrate such ability to sense sugars. Representative absorption and emission spectra for each naphthyl-pyridinium receptor compound with increasing amounts of lactulose in buffer solution are shown in Figs 4 and 5.

Change of absorbance for each receptor compound was monitored as a function of increasing lactulose (Fig. 4). Owing to the nature of the boronic acid charge switch, perturbation of the naphthalene chromophore may occur through the charge-neutralization of the pyridine-boronic acid interaction. Incremental changes of absorbance were observed with increasing amounts of lactulose for each naphthyl-pyridinium receptor compound. Each compound provided absorbance maxima between 300–350 nm and isosbestic points between 330–350 nm for all four receptors. The molar extinction coefficients of 1.17 × 10^4^ (100 µM), 1.10 × 10^4^ (100 µM), 2.77 × 10^4^ (50 µM), and 5.01 × 10^4^ (50 µM) M^−1^ cm^−1^ for **1**–**4**, respectively were obtained. The absorption changes upon sugar binding at physiological pH for each receptor is indicative of a charge transfer process. The effect of lactulose on the fluorescent properties of each receptor compound (**1–4**) examined in phosphate buffer at pH 7.4 is shown as Fig. 5.

Receptor compounds (**1**) and (**2**) exhibited similar emission profiles with emission maxima at 505 and 490 nm. Meanwhile, the emission maxima for (**3**) and (**4**) was 50 nm less, peaking at 440 nm. For these receptor compounds, a minimum of 6-fold increase in intensity was observed in the presence of lactulose at pH 7.4. We found that introduction of lactulose (0.063 mM) provided a drastic intensity change for (**3**) & (**4**) initially, and smaller incremental changes were observed afterward. These types of fluorescent changes have been previously observed by Norrild *et al*., where a pyridinium moiety directly conjugates to an anthracene core^22^. We speculate that the pyridinium unit in these receptor compounds can act as an electron sink for the singlet electron, thus diminishing the fluorescence intensity. Boronic acid-diol recognition and subsequent conversion of the hybridization of boron atom from neutral sp^2^ to anionic sp^3^ generated zwitterion induces an ICT-like mechanism for increasing the fluorescence intensity. It is well understood that the binding properties of boronic acid-based receptors are pH dependent and identifying the pK_a_ of such sensor will provide the optimal window for sugar recognition. Therefore, we determined the pK_a_ of each boronic acid receptor compound (**2**), (**3**), and (**4**) by monitoring the fluorescence-pH in the absence and presence of lactulose (see Supplementary Fig. S1). In the absence of lactulose, the fluorescence intensity of (**2**), (**3**), and (**4**) increased with changing pH resulting in pK_a_ of 7.7, 8.2, and 7.8 respectively. This pK_a_ value shifted to 5.5, 5.8, and 5.7 for (**2**), (**3**), and (**4**) in the presence of lactulose (30 mM), which is indicative of the boronate ester formation from an increased Lewis acidity of the boron atom with sugar binding. Given the higher fluorescence recovery for (**3**) and (**4**) in the presence of lactulose, we examined these boronic acid receptor compounds for other sugar and sugar derivatives to explore potential applications, for example in sugar gut permeability marker. The binding properties of (**3**) and (**4**) provided increased sensitivity (detection limit of 70 and 100 µM) for sugar markers of interest with good dynamic response in the concentration range that is pertinent to sugar gut permeability marker measurements between 100–900 μM (see Supplementary Fig. S2)^39^. These data also suggest other potential application of these standalone-receptor compounds as fluorescent probes to the detection of various types of sugars and sugar derivatives without the need of a reporter dye.

### Formation of a non-fluorescent complex with anionic reporter dyes

**Figure 6:**
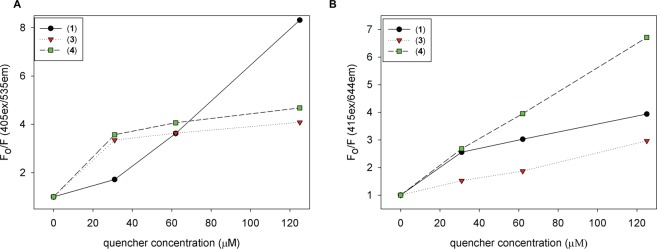
Stern-Volmer plot for the quenching of 4 μM HPTS (**A**) or TSPP (**B**) with each naphthyl-pyridinium quencher compounds (0–125 μM) in 0.1 M sodium phosphate buffer pH 7.4. Excitation and emission wavelengths are given on the axis of graphs.

Given the cationic character of these naphthyl-pyridinium receptor compounds, we sought to investigate their ability to form a non-fluorescent ground state complex with anionic reporter dyes, such as HPTS and TSPP. The fluorescence quenching of each dye can be quantitatively monitored by Stern-Volmer analysis^40^. Two types of quenching can exist: static quenching, due to complex formation, and dynamic quenching, due to collisional encounters between the dye and quencher. Based on previous work with bis-boronic acid-appended viologens (BBV) and their ability to quench HPTS^41^, we reasoned a similar quenching mechanism for each anionic reporter dye through static quenching. Figure 6 shows the effects of adjusting the quencher to dye ratio for HPTS & TSPP with each naphthyl-pyridinium quencher compound, with monitoring the decreasing fluorescence for each dye.

With each quencher having only two cationic groups, we anticipated a need for higher (>400 μM) amounts to achieve at least 80% of quenched fluorescence. However, we found that a concentration of <150 μM (1:38) was needed to achieve ~80% fluorescence. The superior quenching ability likely stems from the conjugated naphthalene core, which can contribute to the formation of a complex with the anionic reporter dyes. The fluorescence data can help to determine quenching efficiency using the Stern-Volmer fitting models, and the best fit to the Stern-Volmer plots for these naphthyl pyridinium quencher compounds was obtained using the sphere of action quenching model (Eq. 1)^42^.

Higher contribution from the static quenching was seen in all conditions that were examined. The higher static quenching constants also reflect this compared to the dynamic constants (Table 1).

**Table 1:** Quenching constants for each naphthyl-pyridinium quencher compounds.

Quencher/Receptor	HPTS *K*_*s*_ (M^−1^)	HPTS *V*(M^−1^)	TSPP *K*_*s*_ (M^−1^)	TSPP *V* (M^−1^)
(1)	1.01 ± 0.5 × 10^5^	63 ± 15	5.24 ± 0.3 × 10^4^	112 ± 46
(3)	9.29 ± 0.4 × 10^4^	109 ± 18	5.01 ± 0.7 × 10^4^	68 ± 13
(4)	1.00 ± 0.9 × 10^5^	100 ± 14	1.45 ± 0.1 × 10^4^	80 ± 10

**Figure 7:**
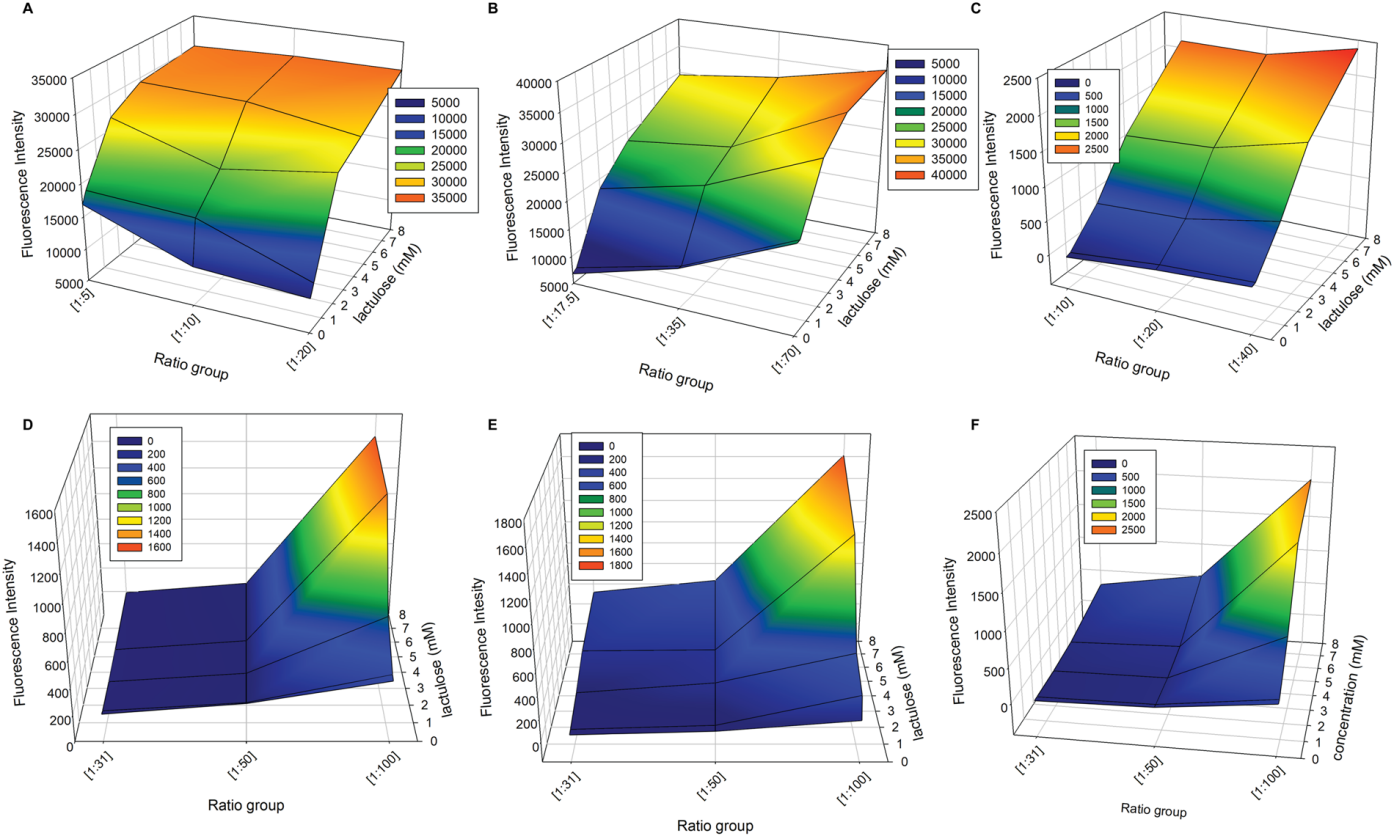
Varying Q:D ratios for optimal signal modulation of each naphthyl-pyridinium receptor compounds for each anionic reporter dye at various amounts of receptor compound to anionic dye in the presence of increasing lactulose (0–8 mM). **A**. HPTS-(**1**) **B**. HPTS-(**3**) **C**. HPTS-(**4**) **D**. TSPP-(**1**) **E**. TSPP-(**3**) **F**. TSPP-(**4**). Each fluorescence recovery measurements were carried out in 0.1 M sodium phosphate buffer pH 7.4 and each dye was 4 μM.

Due to the two-component system’s flexibility, signal modulation can be easily modified by varying the naphthyl-pyridinium receptor concentration against the anionic reporter dye (HPTS or TSPP) while measuring the fluorescence recovery in the presence of an increasing sugar (e.g., lactulose). This method of determining optimal receptor-dye ratio can rapidly identify the limitation of each receptor compound, where it is no longer responsive, and determine the optimal dynamic range as illustrated in Fig. 7.

Simultaneous monitoring of quencher amounts (y-axis) with increasing sugar concentrations (x-axis), highlights the key characteristics of each quencher-dye combination. Notably, each anionic reporter dye has a vastly different π-system, which affects its ability to form a non-fluorescent complex with its respective cationic-quencher. For the HPTS-quencher combinations, a minimal of Q:D = 18:1 for (**3**)- and 20:1 for (**1**)- & (**4**)-HPTS was found to achieve the most dynamic modulation in the presence of lactulose. Additionally, for the TSPP-quencher combinations, minimal Q:D of 100:1 for all three receptor compounds resulted in an optimal dynamic range. While a Q:D of 100:1 for (**3**)-HPTS provided the highest change in fluorescence recovery, it was not a linear or hyperbolic response as usually observed with these quencher-dye combinations; instead it was sigmoidal. Reasons for this difference in quenching character is dependent on the π-system of each anionic dye which could be vastly different, and therefore, interact differently with each cationic receptor compound. For consistency, we pursued compounds combinations that provide linear or hyperbolic response and chose to use 50:1 for (**3**)-HPTS instead.

### Fluorescence recovery of anionic dyes-sugar binding studies

**Figure 8:**
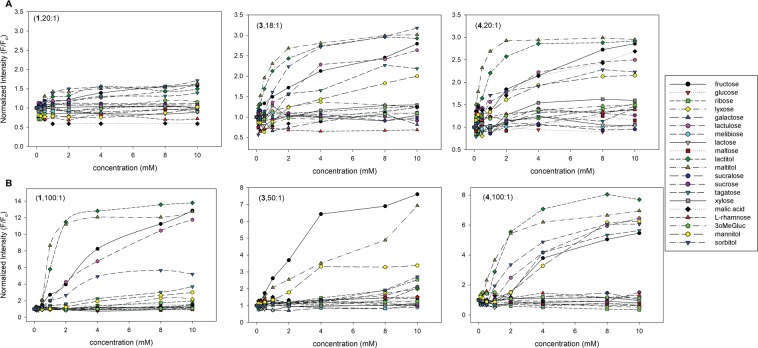
Normalized fluorescent response (**A**) HPTS or (**B**) TPPS combined with each receptor compound in 0.1 M sodium phosphate buffer pH 7.4. F_o_ = initial fluorescence, F = recovered fluorescence after sugar binding. Each dye was used at 4 μM concentration and the amount of each receptor compounds is given in the parentheses of the legend.

Once the optimal combinations of each receptor compound and anionic dye (HPTS or TSPP) was determined, we then examined the binding capabilities of various sugars and sugar derivatives including aldoses, disaccharides, and reducing and non-reducing sugars. With the exclusion of (**2**), because of its lack of cationic and quenching character, the set of 20 sugars were examined with receptors (**1**), (**3**), and (**4**) using HPTS or TSPP anionic dye and the corresponding optimal ratio combinations that were previously established (Fig. 8).

Each boronic acid-appended naphthyl-pyridinium quencher-dye combination provided unique fluorescent recovery characteristics across all 20 sugars, and sugar derivatives were analyzed. We found that (**1**)-HPTS combination exhibited minimal changes for each analyte tested. This was especially clearer that sugars would normally give high recovery (*i.e*., fructose, lactulose, sorbitol, etc), and (**1**) yielded only a 1.5-fold change in recovery. This attributes to the fact that there is only one boronic acid moiety for each neutralized quarternized nitrogen, and for optimal displacement of the anionic HPTS, both charges should neutralize. On the contrary, both (**3**)- and (**4**)-HPTS probes provided good recoveries for at least seven of the twenty sugars examined.

The TSPP-receptor combinations provided significantly different binding characteristics for the examined sugars. All combinations provided between 3- to 13-fold increase in fluorescent signal. For example, (**1**)-TSPP gave good recoveries for lactitol, maltitol, lactulose, fructose, and sorbitol whereas the (**3**)-TSPP probe provided selective fluorescent recovery for only fructose, maltitol, and mannitol. Lastly, the (**4**)-TSPP probe combination gave significant fluorescent recoveries for at least seven of the twenty sugars examined, which is similar to the (**4**)-HPTS probe. Due to the affinity variation of boronic acid for sugars, and also the difference in the non-fluorescent ground-state complex formation, they provide a unique combinatorial approach for the detection of sugars and sugar derivatives in discriminant-based recognition.

## Discussion

We have demonstrated the utility of new boronic acid-appended naphthyl-pyridinium receptors as potential “one-component” and/or “two-component” type chemosensors for the identification and quantitation of various sugars and sugar derivatives. Unlike other one-component boronic acid-appended probes, these compounds can act as both receptors and quenchers for anionic dyes. In their ability to act as one-component probes, sugar recognition induces a charge-neutralization event of the quarternized nitrogen, which in turn, alters the naphthalene moiety chromophore properties, thereby enhancing the fluorescence intensity. We speculate that an ICT-like (ICT “on-off” switch) process upon addition of sugar is the reason for an increased fluorescence. This class of compounds is unique, has not been explored previously, shows the potential to be utilized as standalone chemosensors for sugars and sugar derivatives, and gives an increased sensitivity (2-fold) for lactulose and mannitol. With this increase in sensitivity, the use of these standalone receptors sugar gut marker analysis in gut permeability assessment can be realized. In addition, these receptor compounds were examined for their ability to function in a “two-component” system where each receptor compound is coupled to an anionic dye to form a non-fluorescent ground state complex. The binding profiles for each coupled receptor-dye pair were investigated to determine their performance as a two-component system for the recognition of biologically-relevant sugars and sugar derivatives. Each probe combination provided good fluorescent recoveries for at least seven of the twenty sugars tested. Having a sensing system that offers flexibility in multiple wavelength readings (standalone emission wavelength or coupled reporter dye wavelength) can be advantageous especially when analyzing samples gut markers in complex media such as urine for gut permeability assessment. The chemosensors can be readily implemented in an array-based recognition platform for differential based recognition alleviating potential signal interference from urine species. Further studies are underway to elucidate the fluorescence mechanism of each boronic acid receptor compound and devise methods for enhancing their fluorescent properties.

## Materials and Methods

### Materials

All chemicals and solvents were purchased from Sigma-Aldrich or Fisher Scientific and used as received. Bromomethylphenyl boronic acid was purchased from Combiblocks. 3,5-bisbromomethyl phenylboronic acid was synthesized according to the previously published procedure^41^. Naphthyl pyridine starting materials were synthesized via bipyridyl Suzuki-Miyaura coupling procedure.

### Synthesis of boronic acid appended naphthyl pyridinium receptor compounds

Naphthyl pyridines were subjected to nucleophilic substitution with 3,5-bisbromomethylphenyl boronic acid or bromomethyl phenylboronic acid to create the quarternized product in good yields. ^1^H-, ^13^C-NMR spectra characterized all of the products and MS. Detailed synthetic procedures and spectra are given in the Supplementary Information (SI).

### Absorption measurements of boronic acid appended naphthyl-pyridinium compounds

Agilent Technologies Cary 60 Vis instrument performed all absorption measurements in a quartz cuvette (1 cm pathlength). Stock solutions (1 mg/mL) of each compound was initially prepared in DMSO and then diluted to the desired concentration in 0.1 M sodium phosphate buffer pH 7.4, which gave minimal DMSO amount of at least 4% v/v. Concentrations for each boronic acid-appended naphthyl-pyridinium receptors in cuvette are as follows; (**1**) = 100 μM, (**2**) = 100 μM, (**3**) = 50 μM, and (**4**) = 50 μM. Measurements were done *in situ* by taking the absorbance of each receptor compound in phosphate buffer pH 7.4, then aliquots of buffered 1 M lactulose (0.5–7 μL) was added, shaken for 30 seconds, and the new absorbance was recorded.

### Fluorescence Measurements of boronic acid appended naphthyl-pyridinium compounds

Stock solutions of each boronic acid receptor compound (**1–4**) were initially prepared in DMSO (1 mg/mL) and then diluted to the desired initial 1 mM (for 1 & 2) or 0.5 mM (for 3 & 4) concentration before being added to the wells. Each lactulose concentration point was prepared in 0.1 M sodium phosphate buffer at 2-fold the desired final concentration in each well. Each well received 20 µL of receptor compound and sugar concentration, (each data point recorded in triplicates), and the fluorescence measurements were conducted in a 96-well plate (Corning #3694) using a Tecan Infinite M1000 instrument, a plate reader (gain 100, flashes 30, z-position 1.8 cm). Plates were shaken for 30 seconds (2 mm orbital amplitude) before reading.

### Stern-Volmer quenching of HPTS and TSPP anionic reporter dyes

Stock solutions of each boronic acid quencher compound (**1**, **3**, and **4**) were initially prepared in DMSO (1 mg/mL) and then diluted in 0.1 M sodium phosphate buffer pH 7.4 to obtain the desired initial 2-fold concentration. Fluorescence measurements were conducted in a 96-well plate (Corning #3694), and to each well, 20 µL of each quencher compound and 8 µM of HPTS or TSPP were added in triplicate. Initial fluorescence wells contained 20 µL of reporter dye and 20 µL of buffer. Blank wells received 40 µL of buffer only. Measurements were performed using a Tecan Infinite M1000 instrument, a plate reader (gain 100, flashes 30, z-position 1.8 cm) Plates were shaken for 30 seconds (2 mm orbital amplitude) prior to reading. After blank subtraction, fluorescence intensity relative to initial total fluorescence of HPTS or TSPP (F_o_/F) for each sample was calculated.

### Fluorescence recovery of each anionic reporter dyes

Fluorescence recovery measurements for sugar binding studies were conducted by preparing 96-well plates with the addition 20 µL of the naphthyl pyridinium-dye (HPTS or TSPP) probe solution as 2-fold concentrate (for *e.g*., 160 µM for (**1**) & 8 µM HPTS or 800 µM for (**1**) & 8 µM for TSPP) in 0.1 M sodium phosphate buffer pH 7.4. At the time of running the assay, each well received 20 µL of sample performed in triplicate. Blank wells were given 40 µL buffer with neither HPTS nor boronic acid receptor compound. Baseline fluorescence wells contained 20 µL of buffer and 20 µL of probe solution. Fluorescence recovery of HPTS was measured on the plate reader. After blank subtraction, fluorescence intensity relative to initial quenched HPTS (F/F_o_) for each sample was calculated. Under these conditions, F_o_ is a non-zero value after background subtraction with about 20% of maximum fluorescence intensity.

### Data analysis

Stern-Volmer quenching constants were determined by non-linear curve fitting using the following eq. (1)^41,43^.

1 $$\frac{{F}_{o}}{F}=(1+{K}_{s}[Q]{e}^{V[Q]}$$

Where *V* is the dynamic quenching constant, *K*_*s*_ is the static quenching constant, [Q] is the concentration of the quencher, and e^*V*[Q]^ is derived from the Poisson distribution. *K* was solved using OriginLab software (OriginLab Corp, Northampton, MA, USA).

## Supplementary information

Supplementary Information
