# Supplementary material for: Boronic Acid Appended Naphthyl-Pyridinium Receptors as Chemosensors for Sugars
Source: Sci Rep. 2019 Apr 30;9:6651. doi: 10.1038/s41598-019-42812-8 (PMC6491427; doi:10.1038/s41598-019-42812-8)
Supplement: Supplementary file 1 — Supplementary Information [file 41598_2019_42812_MOESM1_ESM.pdf]

Supporting Information

Boronic Acid Appended Naphthyl-Pyridinium Receptors as Chemosensors for Sugars

Angel Resendez[1], Sanjay V. Malhotra[1,2]

Departments of [1]Radiation Oncology and [2]Radiology, Stanford University School of Medicine,

Stanford, California 94305, United States

1. Synthesis of boronic acid appended naphthyl-pyridinium receptors p. S-2–S-5
2. pH profile and binding characteristics of boronic acid receptors p. S-6–S-9
3. ^{1}H and ^{13}C NMR spectra (Fig. S-3–S-18) p.S-10–S-25
4. References p.S-26

1. Synthesis

The synthesis of each boronic acid appended naphthyl-pyridinium receptor compounds was obtained via Suzuki-Miyaura coupling using a modified version of previously reported procedures followed by nucleophillic substitution with the appropriate bromomethyl phenyl boronic acid. The synthesis of 2-(3,5-bis(bromomethyl)phenyl)-1,3,2-dioxaborinane was followed according to published procedure.[1]

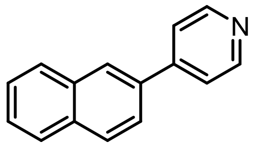

Synthesis of 4-naphthalenyl pyridine-(a)[2]

In a 3-neck round bottom flask (25 mL) purged with nitrogen, a solution containing tetrakis (triphenylphosphine) palladium (0.034 g, 0.03 mmol), CsCO3 (0.423 g, 1.2 mmol), and 4-pyridinyl boronic acid (0.147 g, 1.2 mmol) in DMF (7 mL) was mixed and allowed to stir for 30 minutes at 25 °C. 1-bromonaphthalene (0.140 mL, 1mmol) was added and the mixture stirred at 100 °C for 24 h. After cooling to room temperature, the mixture was washed with deionized water (30 mL) and extracted with DCM (3 x 15 mL). The organic fraction was dried with $MgSO_4$ and concentrated in a slurry of silica and further purified by flash chromatography with a mixture 2:1 of Hex:EtOAc/EtOH(3:1) as eluent to give the title compound as a white amorphous solid (0.131 g, 64% yield). ^{1}H NMR (400 MHz, Chloroform-*d*) δ 8.77 – 8.70 (m, 2H), 8.00 – 7.90 (m, 2H), 7.94 – 7.80 (m, 2H), 7.55 (dd, *J* = 8.3, 7.0 Hz, 2H), 7.55 – 7.43 (m, 2H), 7.48 – 7.38 (m, 4H); ^{13}C NMR (101 MHz, Chloroform-*d*) δ 150.33, 149.81, 148.66, 137.34, 133.74, 130.72, 128.84, 128.50, 126.84, 126.62, 126.15, 125.32, 125.15, 125.01, 121.83. MS-ESI m/z calculated for $C_{15}H_{11}N$ $[M+H]^+$: 206.093, found 206.

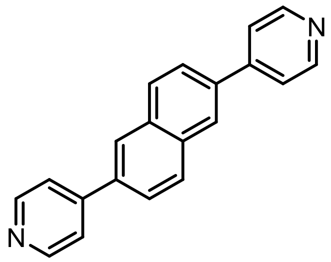

Synthesis of 2,6-dipyridinyl naphthalene-(b)[3]

To an oven dried 3-neck round bottom flask (25 mL) purged with argon, was added $Pd(OAc)_2$ (0.018 g, 0.08 mmol, 4 mol%), XPhos (0.046 g, 0.096 mmol, 4.8 mol%), 2,6-dibromonaphthalene (0.570 g, 2 mmol), 4-pyridinyl boronic acid (0.540 g, 4.4 mmol), and 1,4-dioxane (11.2 mL). After the mixture pre-stirred at 25 °C for 30 minutes, a degassed aqueous solution of NaOH (2.8 mL, 10.2 mmol, 1.2 M) was added to the mixture and vigorously stirred at 95°C for 48 h. At the end of the reaction, the organics were extracted with ethyl acetate (2 x 10

mL) and subsequently washed with deionized water (10 mL x 2). The organic dried with $MgSO_4$ and concentrated on a rotary evaporator. A silica slurry was made and was purified by silica gel flash chromatography with a mixture 3:2 of Hex:EtOAc/EtOH(3:1) as eluent to give the title compounds as a white crystalline solid (0.338g, 59% yield). ^{1}H NMR (400 MHz, Chloroform-*d*) δ 8.78 – 8.70 (m, 4H), 8.19 – 8.14 (m, 2H), 8.05 (d, *J* = 8.5 Hz, 2H), 7.82 (dd, *J* = 8.5, 1.7 Hz, 2H), 7.69 – 7.62 (m, 4H); ^{13}C NMR (101 MHz, $CDCl_3$) δ 150.42, 147.88, 136.37, 133.45, 129.49, 126.21, 125.43, 121.80. MS-ESI m/z calculated for $C_{20}H_{14}N_2$ $[M+H]^+$: 283.119, found 283.

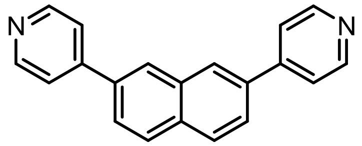

Synthesis of 2,7-dipyridinyl naphthalene-(c)

To an oven dried 3-neck round bottom flask (25 mL) purged with nitrogen, was added $Pd(OAc)_2$ (0.018 g, 0.08 mmol, 4 mol%), XPhos (0.046 g, 0.096 mmol, 4.8 mol%), 2,7-dibromonaphthalene (0.570 g, 2 mmol), 4-pyridinyl boronic acid (0.540 g, 4.4 mmol), and 1,4-dioxane (11.2 mL). After the mixture pre-stirred at 25 °C for 30 minutes, a degassed aqueous solution of NaOH (2.8 mL, 10.2 mmol, 1.2 M) was added to the mixture and vigorously stirred at 95°C for 48 h. At the end of the reaction, the organics were extracted with ethyl acetate (2 x 10 mL) and subsequently washed with deionized water (10 mL x 2). The organic dried with $MgSO_4$ and concentrated on a rotary evaporator. A silica slurry was made and was purified by silica gel flash chromatography with a mixture 3:2 of Hex:EtOAc/EtOH(3:1) as eluent to give the title compounds as a white crystalline solid (0.30 g, 53% yield). ^{1}H NMR (400 MHz, Chloroform-*d*) δ 8.76 – 8.70 (m, 4H), 8.20 (dt, *J* = 1.6, 0.7 Hz, 2H), 8.01 (dt, *J* = 8.7, 0.8 Hz, 2H), 7.81 (dd, *J* = 8.6, 1.8 Hz, 2H), 7.69 – 7.61 (m, 4H), 7.57 – 7.50 (m, 1H); ^{13}C NMR (101 MHz, Chloroform-*d*) δ 150.69, 150.42, 147.90, 136.34, 133.56, 133.34, 128.79, 126.88, 125.54, 121.80, 121.37; MS-ESI m/z calculated for $C_{20}H_{14}N_2$ $[M+H]^+$: 283.119, found 283..

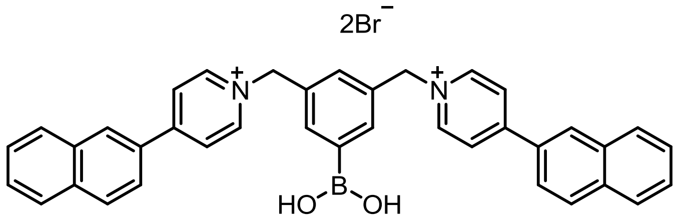

Synthesis of 1,1'-(5-borono-1,3-phenylene)bis(methylene)bis(4-naphthalenyl)pyridinium-(1)

Naphthyl pyridine (0.513 g, 2.5 mmol) was added to a solution of 3,5-bis(bromomethyl)phenyl)-dioxaborinane (0.347 g, 1 mmol) in DMF and stirred at 65 °C for 48 h. To the lightly yellow solution, cold acetone was added (20 mL) to induce precipitation of a white solid. The solid was

collected by centrifugation and was washed with acetone. To deprotect the boronic acids, the solid was dissolved in methanol (~10 mL), 12 M HCl (0.5 mL) and heated at 80 °C for 30 min. After cooling to room temperature, the mixture was concentrated and cold acetone was added and stored at 4 °C for 2 h. The solid was then collected, washed with acetone, and dried under a stream of nitrogen to afford a white solid (0.524 g, 73% yield). ^{1}H NMR (400 MHz, Methanol-*d*$_4$) δ 9.26 – 9.11 (m, 4H), 8.33 – 8.24 (m, 4H), 8.13 – 7.86 (m, 9H), 7.70 – 7.52 (m, 8H), 6.03 (s, 4H); ^{13}C NMR (101 MHz, Methanol-*d*$_4$) δ 158.32, 144.44, 134.07, 133.95, 133.42, 131.29, 129.60, 129.04, 128.70, 128.37, 127.66, 126.59, 125.16, 123.52, 63.27. MS-ESI m/z calculated for $C_{22}H_{19}BNO_2$ [M-2Br]$^+$: 340.151, found 340.200.

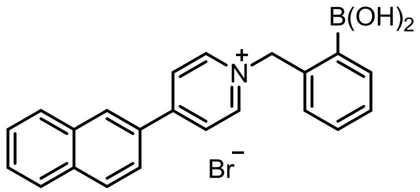

Synthesis of 1-(2-boronobenzyl)-4-(naphthalen-2-yl) pyridinium bromide-(**2**)

To a solution of 2-bromomethylphenyl boronic acid (0.3 g, 3.6 mmol) in DMF (10 mL) was added, 4-naphtyl pyridine (0.846 g, 3 mmol), and the reaction was stirred at 65 °C for 48 hours. The reaction mixture was cooled to room temperature DCM (25 mL) and the organics were washed with deionized water (3 x 15 mL) and set aside. The aqueous fraction was frozen and the water was removed via lyophilization. Yellow solid was obtained and subsequently washed with cold acetone before collecting. The solid was centrifuged, collected, and dried under a stream of nitrogen (0.611 g, 48% yield). ^{1}H NMR (400 MHz, DMSO-*d*$_6$) δ 9.16 – 9.09 (m, 2H), 8.36 – 8.29 (m, 2H), 8.20 – 8.06 (m, 2H), 7.93 (s, 1H), 7.87 – 7.79 (m, 2H), 7.72 – 7.57 (m, 3H), 7.55 – 7.41 (m, 2H), 6.10 (s, 2H); ^{13}C NMR (101 MHz, DMSO-*d*$_6$) δ 156.70, 145.08, 138.12, 135.85, 134.07, 133.77, 131.40, 130.96, 130.36, 129.77, 129.30, 129.11, 128.94, 128.88, 128.24, 127.29, 126.09, 124.51, 63.43. MS-ESI m/z calculated for $C_{22}H_{19}BNO_2$ [M-Br]$^+$: 340.151, found 340.200.

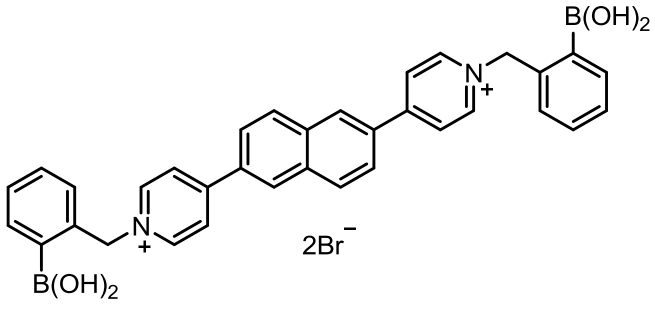

Synthesis of 4,4'-(naphthalene-2,6-diyl)bis(1-(2-boronobenzyl)pyridinium-(3)

To a solution of 2-bromomethylphenyl boronic acid (0.451 g, 2.1 mmol) in DMF (7 mL) was added, 2,6-dipyridinyl naphthalene (0.282 g, 1 mmol), and the reaction was stirred at 65 °C for 48 hours. The reaction mixture was cooled to room temperature and cold acetone (25 mL) was added to induce further precipitation of a pale yellow solid. The precipitate was centrifuged, washed with acetone (3 x 20 mL) and dried under a stream of nitrogen (0.571 g, 79% yield). ^{1}H NMR (400 MHz, DMSO-d_6) δ 9.16 – 9.09 (m, 4H), 8.86 (d, *J* = 1.8 Hz, 2H), 8.73 – 8.64 (m, 4H), 8.52 (s, 4H), 8.32 (d, *J* = 8.7 Hz, 2H), 8.25 (dd, *J* = 8.7, 1.8 Hz, 2H), 7.86 – 7.78 (m, 2H), 7.53 – 7.39 (m, 4H), 7.32 (dd, *J* = 7.7, 1.4 Hz, 2H), 6.05 (s, 4H); ^{13}C NMR (101 MHz, DMSO-d_6) δ 154.68, 145.48, 135.86, 133.51, 131.07, 130.91, 129.80, 129.39, 128.82, 126.03, 125.40, 63.29. MS-ESI m/z calculated for $C_{34}H_{30}B_2N_2O_4$ $[M+H]^+$: 551.253, found 551.200.

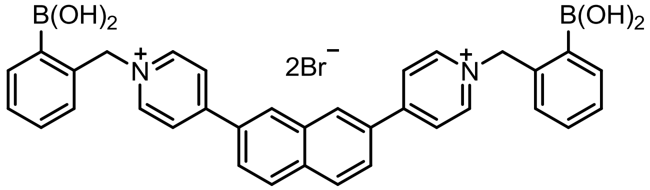

Synthesis of 4,4'-(naphthalene-2,7-diyl)bis(1-(2-boronobenzyl)pyridinium-(4)

To a solution of 2-bromomethylphenyl boronic acid (0.473 g, 2.2 mmol) in DMF (7 mL) was added, 2,7-dipyridinyl naphthalene (0.282 g, 1.1 mmol), and the reaction was stirred at 65 °C for 48 hours. The reaction mixture was cooled to room temperature and cold acetone (25 mL) was added to induce further precipitation of a pale yellow solid. The precipitate was centrifuged, washed with acetone (3 x 20 mL) and dried under a stream of nitrogen (0.685 g, 87% yield). ^{1}H NMR (400 MHz, DMSO-d_6) δ 9.12 (d, *J* = 6.3 Hz, 4H), 8.85 (d, *J* = 3.3 Hz, 2H), 8.68 – 8.60 (m, 4H), 8.52 (bs, 4H), 8.33 – 8.24 (m, 4H), 7.87 – 7.77 (m, 2H), 7.55 – 7.40 (m, 4H), 7.33 (d, *J* = 7.5 Hz, 2H), 6.05 (s, 4H); ^{13}C NMR (101 MHz, DMSO-d_6) δ 154.75, 145.51, 138.32, 135.88, 135.75, 133.09, 132.61, 130.92, 130.55, 129.89, 128.84, 127.22, 125.31, 63.27. MS-ESI m/z calculated for $C_{34}H_{30}B_2N_2O_4$ $[M+H]^+$: 551.253, found 551.200.

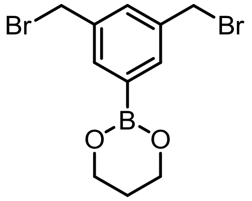

Synthesis of 2-(3,5-bis(bromomethyl)phenyl)-1,3,2-dioxaborinane

To a 100 mL round bottom flask fitted with a condenser and a sidearm was added 3,5-dimethyl phenyl boronic acid (1.5 g, 10 mmol), calcium hydride (0.843 g, 20 mmol), and dichloroethane (50 mL). After 10 minutes of stirring under nitrogen, 1,3 propanediol (0.80 mL, 11 mmol) was added via syringe. The reaction was refluxed for 2 h, cooled to room temperature, and filtered. The filtrate was mixed with N-bromosuccinimide (3.91g, 22 mmol) and 2,2'-

azobisisobutryonitrile (0.328 g, 2 mmol) and refluxed for 3 h. The orange solution was cooled overnight, and the succinate crystals were filtered off. The filtrate was concentrated leaving an off-white solid, which was recrystallized from methanol (1.5 g, 45% yield). ^{1}H NMR (400 MHz, Chloroform-*d*) δ 7.72 (d, *J* = 1.9 Hz, 2H), 7.47 (t, *J* = 1.9 Hz, 1H), 4.48 (s, 4H), 4.21 – 4.14 (m, 4H), 2.04 (q, *J* =5.5 Hz 2H); ^{13}C NMR (101 MHz, Chloroform-*d*) δ 137.56, 134.33, 131.73, 62.04, 33.15, 27.36.

2. pH profile and binding characteristics

Monitoring of the pH profiles for each boronic acid receptor in the absence or presence of lactulose

Titration curves with pH were determined the following buffer solutions pH 3-4 acetate buffer was used; pH 5-8.5 phosphate buffer; and pH 9-10 carbonate buffer. Fluorescence intensity was monitored in the absence or presence of 30 mM lactulose for (**2**, 500 µM), (**3**, 250 µM) and (**4**, 205 µM) receptor compounds in indicated buffer conditions. Stock solutions of each boronic acid receptor compound (**2**, **3,** and **4**,) were initially prepared in DMS (1 mg/mL) and then diluted in appropriate buffer conditions obtain the desired initial 2-fold concentration. Fluorescence measurements were conducted in a 96-well plate (Corning #3694) and to each well 30 µL of each receptor compound and buffer media with or without lactulose were added in triplicate. Blank wells received 60 µL of buffer only. Measurements were performed using a Tecan Infinite M1000 instrument, a plate reader (gain 100, flashes 30, z-position 1.8 cm) Plates were shaken for 30 seconds (2 mm orbital amplitude) prior to reading. After blank subtraction, fluorescence intensity relative to initial total fluorescence (at pH 3)

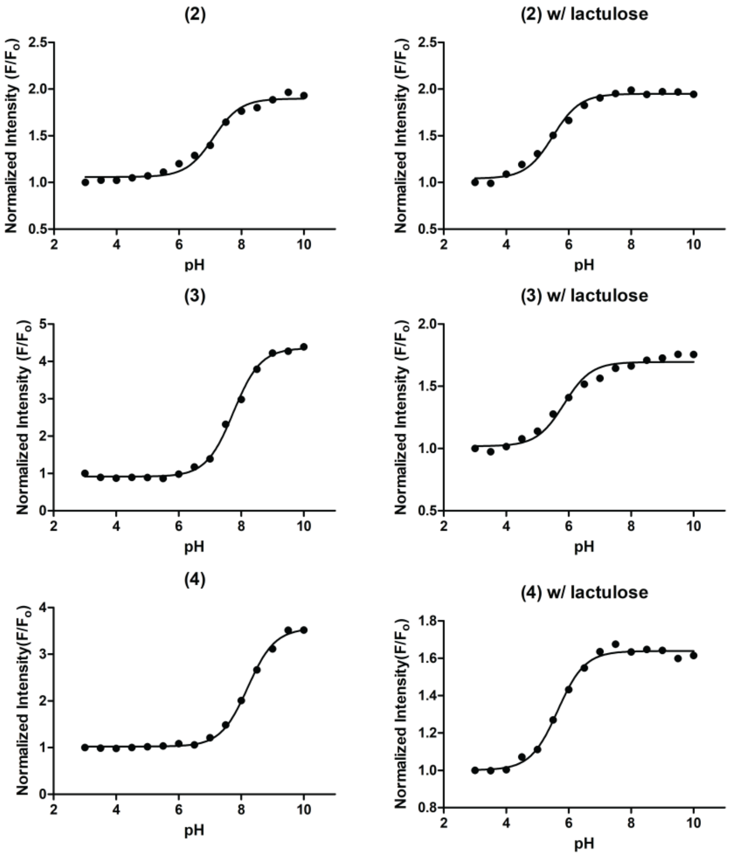

Figure S1. pH profiles of (**2**), (**3**), and (**4**) generated via fluorescence spectroscopy in different buffer conditions in the absence or presence of 30 mM lactulose.

Each pH-profile was fitted with equation S1, where y= normalized intensity, y_{max}= maximum normalized intensity, y_{min}=minimum normalized intensity, x= pH value, k= acid dissociation constant and pk_a values were obtained from this constant (pk_a= -log k).

$$y = \frac{y_{\max} + y_{\min} 10^{(x-k)}}{(1+10^{(x-k)})} \quad \text{(Eq. S1)}$$

Table S1. pK_a values determined by fluorescence spectroscopy of each boronic acid receptor compound in the absence or presence of lactulose in different pH conditions. Titration curves fitted to equation S1.

Boronic acid Receptor (µM)	pK_a-absence of lactulose	pk_a-with 30 mM lactulose	R^2
**2** (500 µM)	7.7 ± 0.08	5.5 ± 0.06	0.989, 0.984
**3** (250 µM)	7.8 ± 0.03	5.8 ± 0.10	0.997, 0.978
**4** (250 µM)	8.2 ± 0.02	5.7 ± 0.05	0.998, 0.994

Saturation binding curves of each boronic acid receptor compound

Fluorescence intensity was monitored in the of increasing sugar concentration (0-10 mM) for (**3**, 250 µM) and (**4**, 250 µM) receptor compounds in 0.1 M sodium phosphate buffer, pH 7.4. Stock solutions of each boronic acid receptor compound (**3,** and **4**,) were initially prepared in DMS (1 mg/mL) and then diluted in sodium phosphate buffer obtain the desired initial 2-fold concentration. Fluorescence measurements were conducted in a 96-well plate (Corning #3694) and to each well 30 µL of each receptor compound and sugar in buffer media was added in triplicate. Blank wells received 60 µL of buffer only. Baseline (Fo) well received receptor and buffer media. Measurements were performed using a Tecan Infinite M1000 instrument, a plate reader (gain 100, flashes 30, z-position 1.8 cm) Plates were shaken for 30 seconds (2 mm orbital amplitude) prior to reading. After blank subtraction, fluorescence intensity relative to initial baseline fluorescence was plotted with respect to increasing sugar concentration (Figure S2).

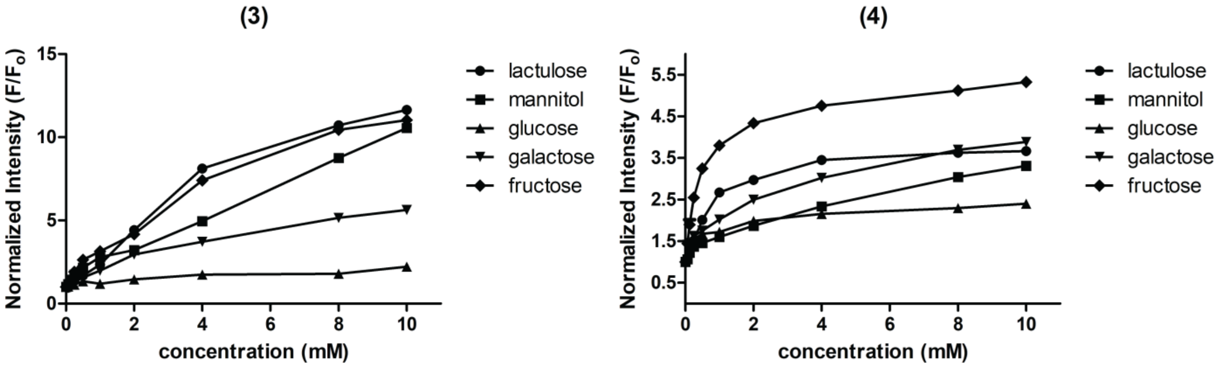

Figure S2. Normalized fluorescence response of (**3**) and (**4**) in the presence of lactulose, mannitol, glucose, galactose, or fructose in 0.1 M sodium phosphate buffer pH 7.4. Excitation wavelength used λ_{ex}= 340 nm and emission λ_{em} = 435nm for each receptor compound.

Table S-2. Apparent affinity constants (K_b, M^{-1}) for each boronic acid appended napthyl-pyrdinium receptor (**3**) and (**4**) for the 4 sugars studied. The limits of detection (LOD) and quantification (LOQ) were defined as the analyte concentration in which the fluorescence intensity in the assay was 3 and 10 standard deviations above the mean baseline fluorescence

Sugar/Boronic acid receptor	Lactulose	Mannitol	Glucose	Galactose	Fructose	LOD/LOQ (µM)
(**3**)	1042 ± 102	189 ± 49	17 ± 3	473 ± 112	1983 ± 145	100/200
(**4**)	270±30	13±5	16±9	19±6	315±20	70/190

Apparent stability constants were determined by non-linear curve fitting using equation S2.

$$\frac{F}{F_o} = \frac{\left(1 + \frac{F_{\max}}{F_o}\right) K_b[A]}{1 + K_b[A]}$$

Eq. S2

where F_0 is the fluorescence intensity of the quenched dye, F is the fluorescence intensity after the addition of analyte, F_{max} is the fluorescence intensity at which no further signal is obtained with further analyte addition, K_b is the apparent stability constant, and [A] is analyte concentration. K_b was solved using OriginLab software (Originlab Corp, Northampton, MA, USA).

3. NMR Spectra

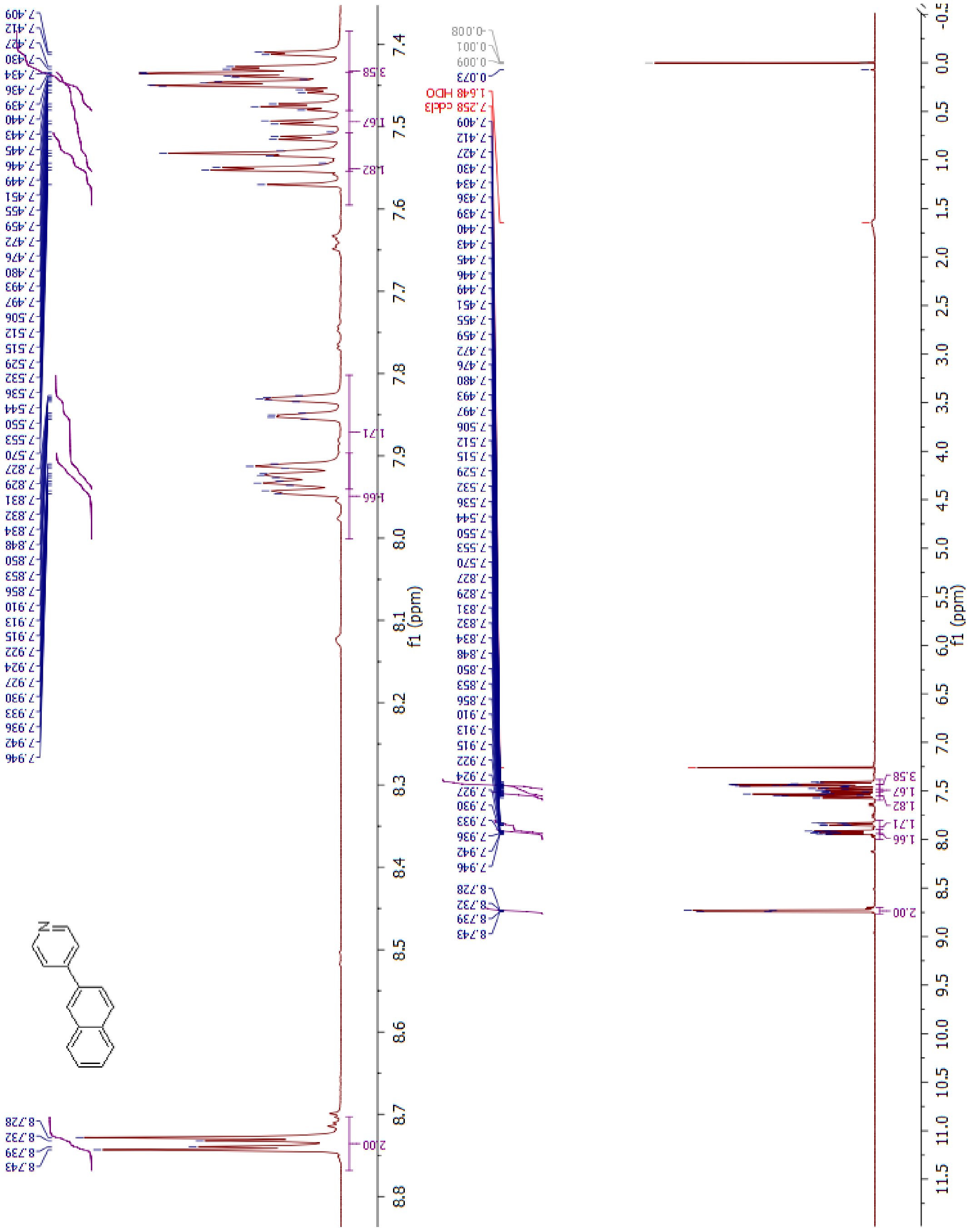

Figure S-3. ^{1}H-NMR spectra of (a)

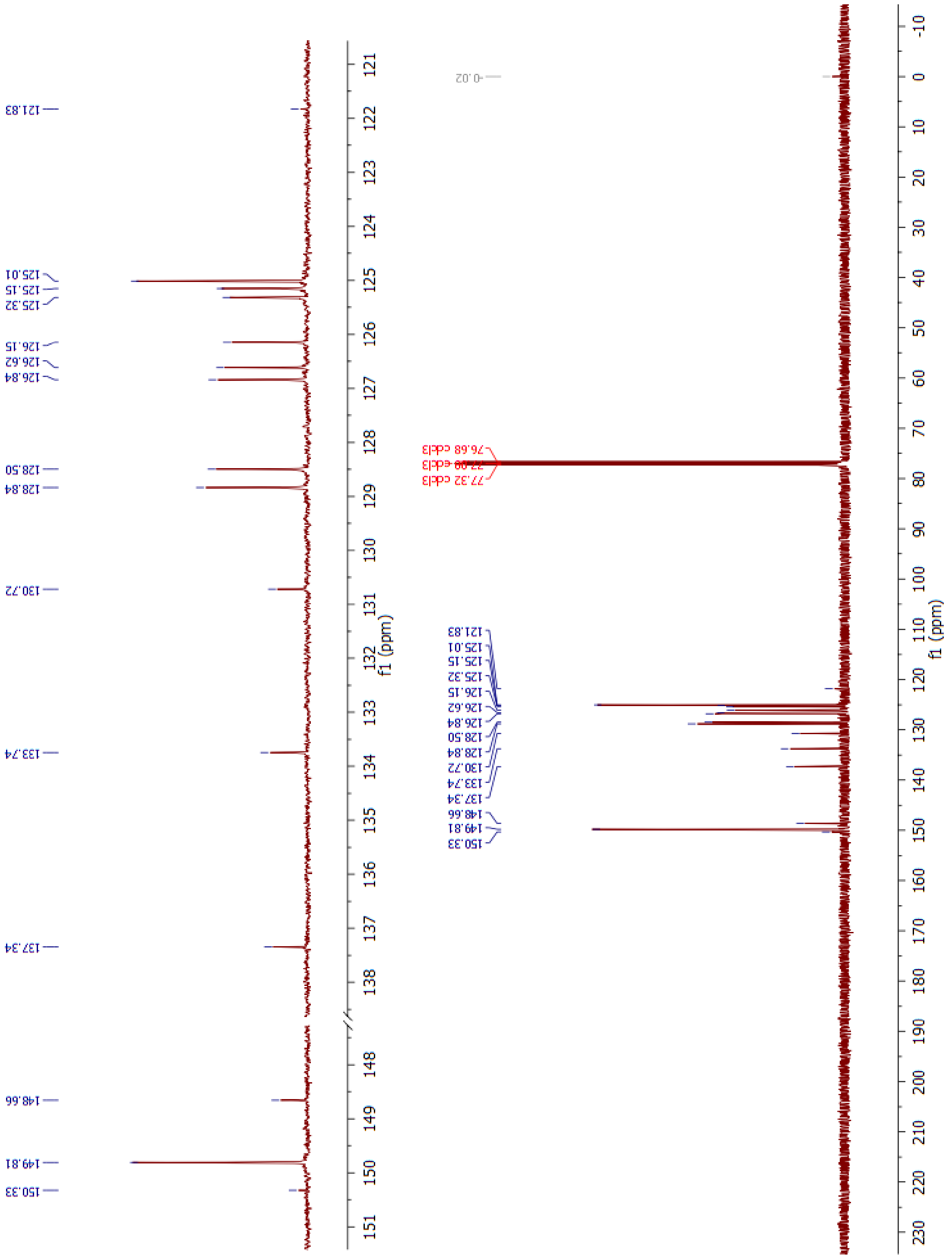

Figure S-4. ^{13}C-NMR spectra of (a)

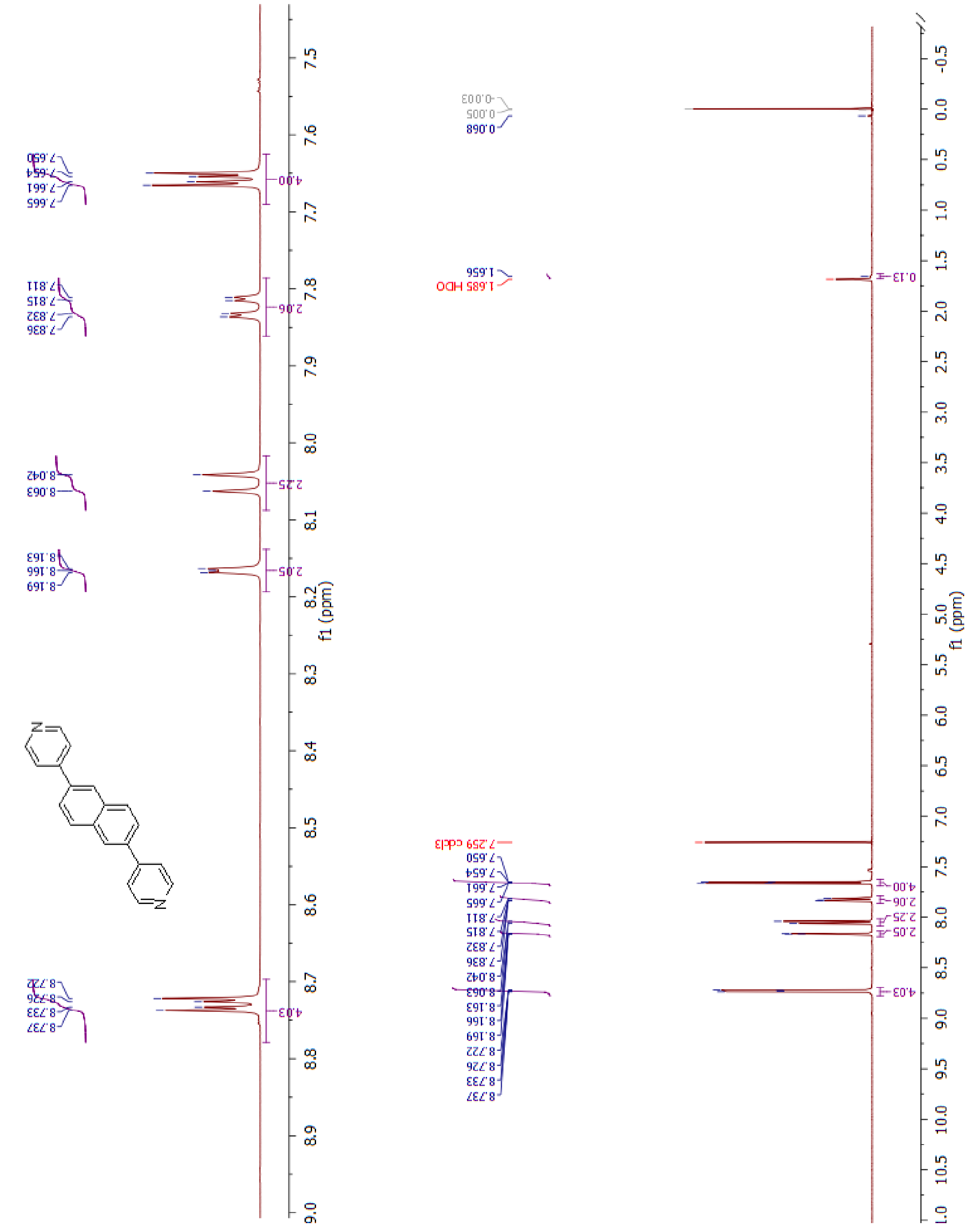

Figure S-5. ^{1}H-NMR spectra of (b)

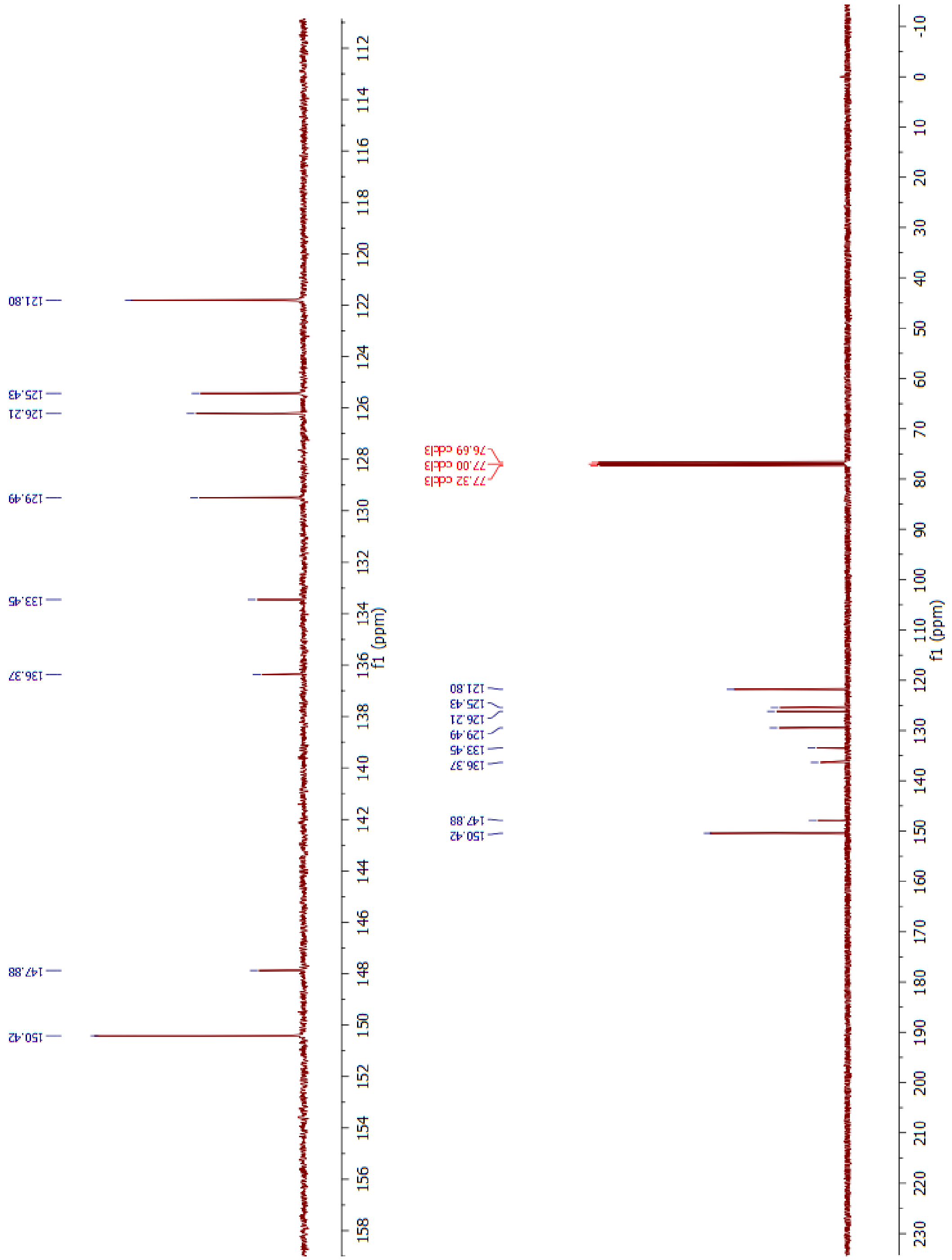

Figure S-6. ^{13}C-NMR spectra of (b)

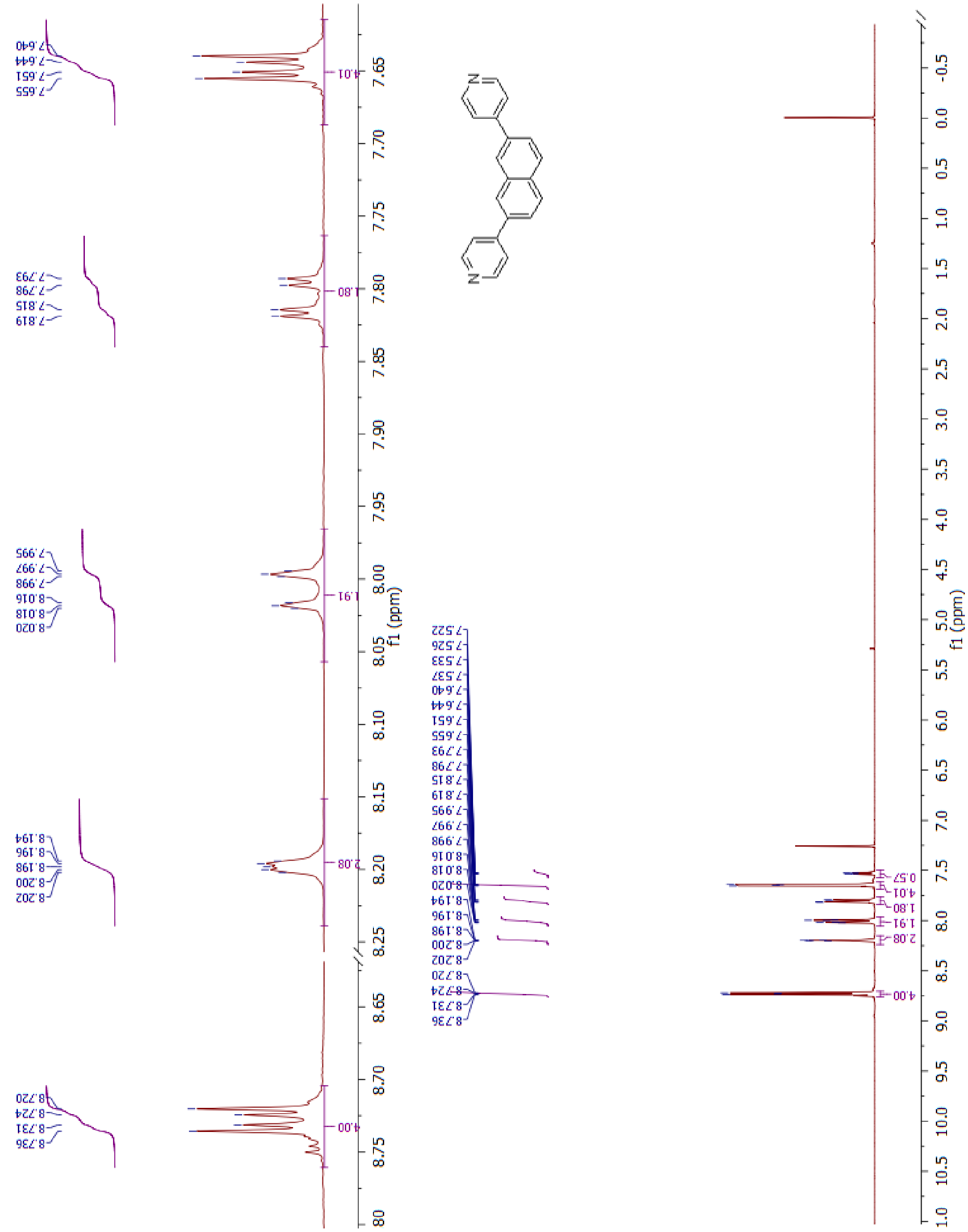

Figure S-7. ^{1}H-NMR spectra of (c)

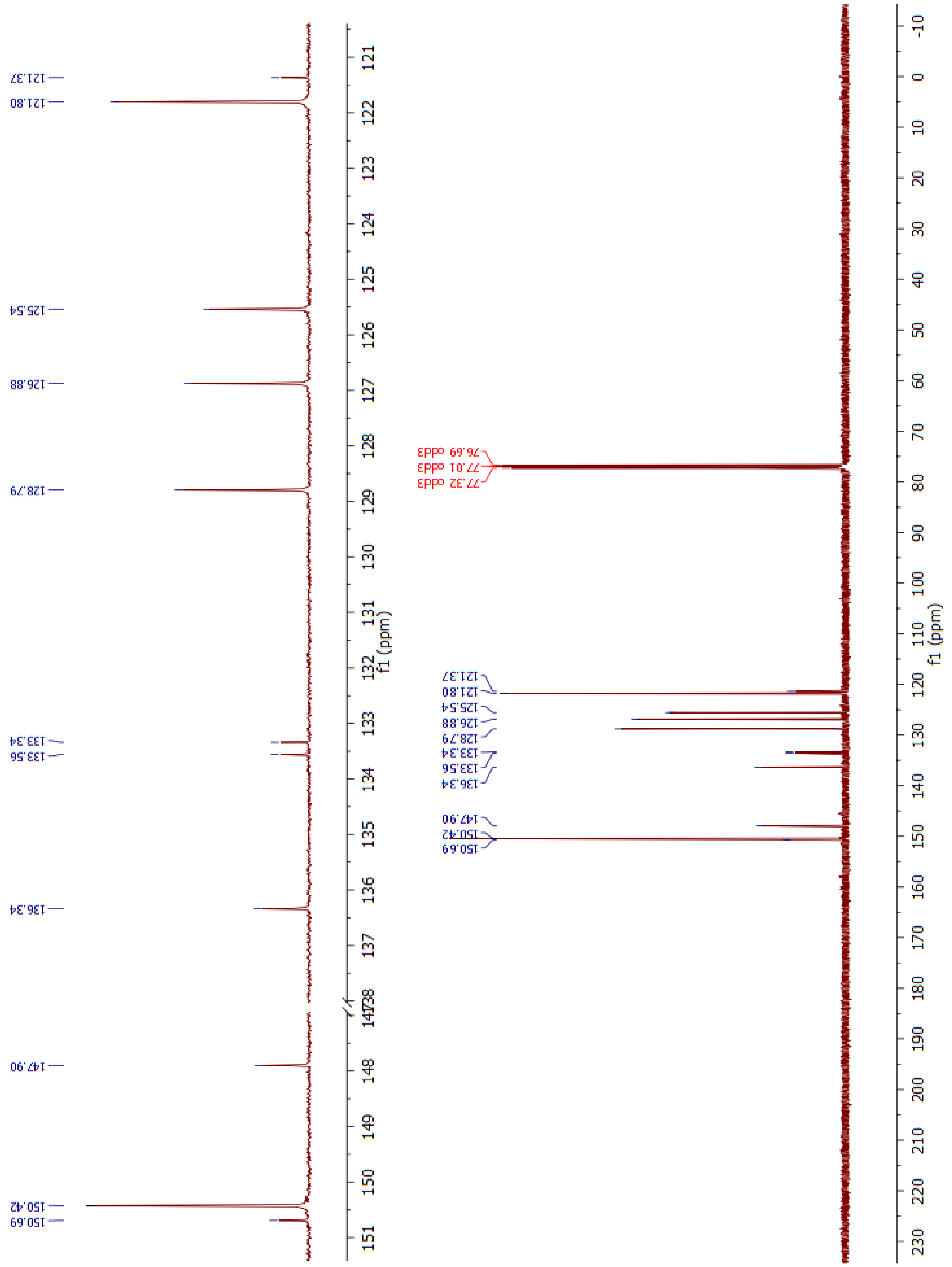

Figure S-8.^{13}C-NMR spectra of (c).

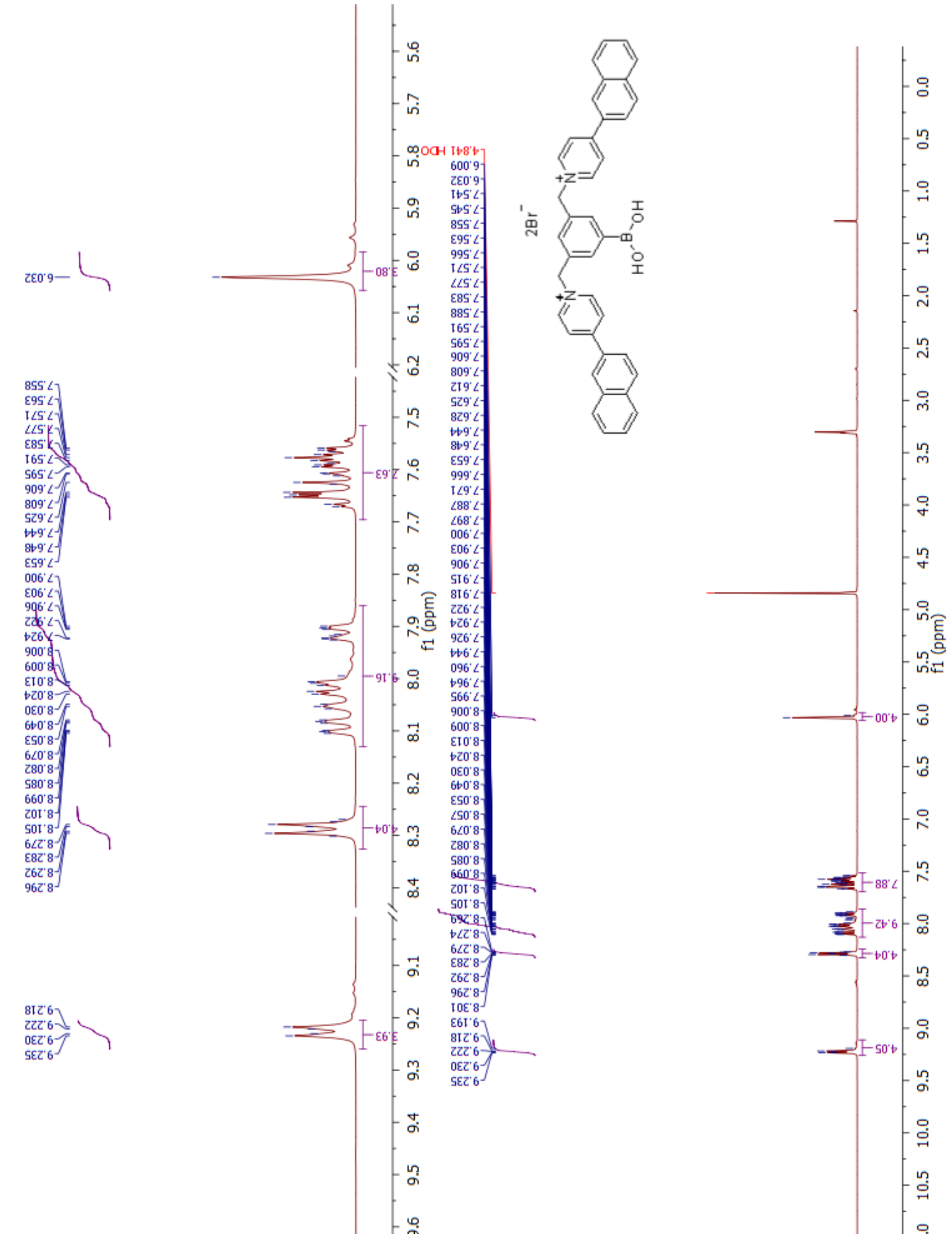

Figure S-9.^{1}H-NMR spectra of (**1**)

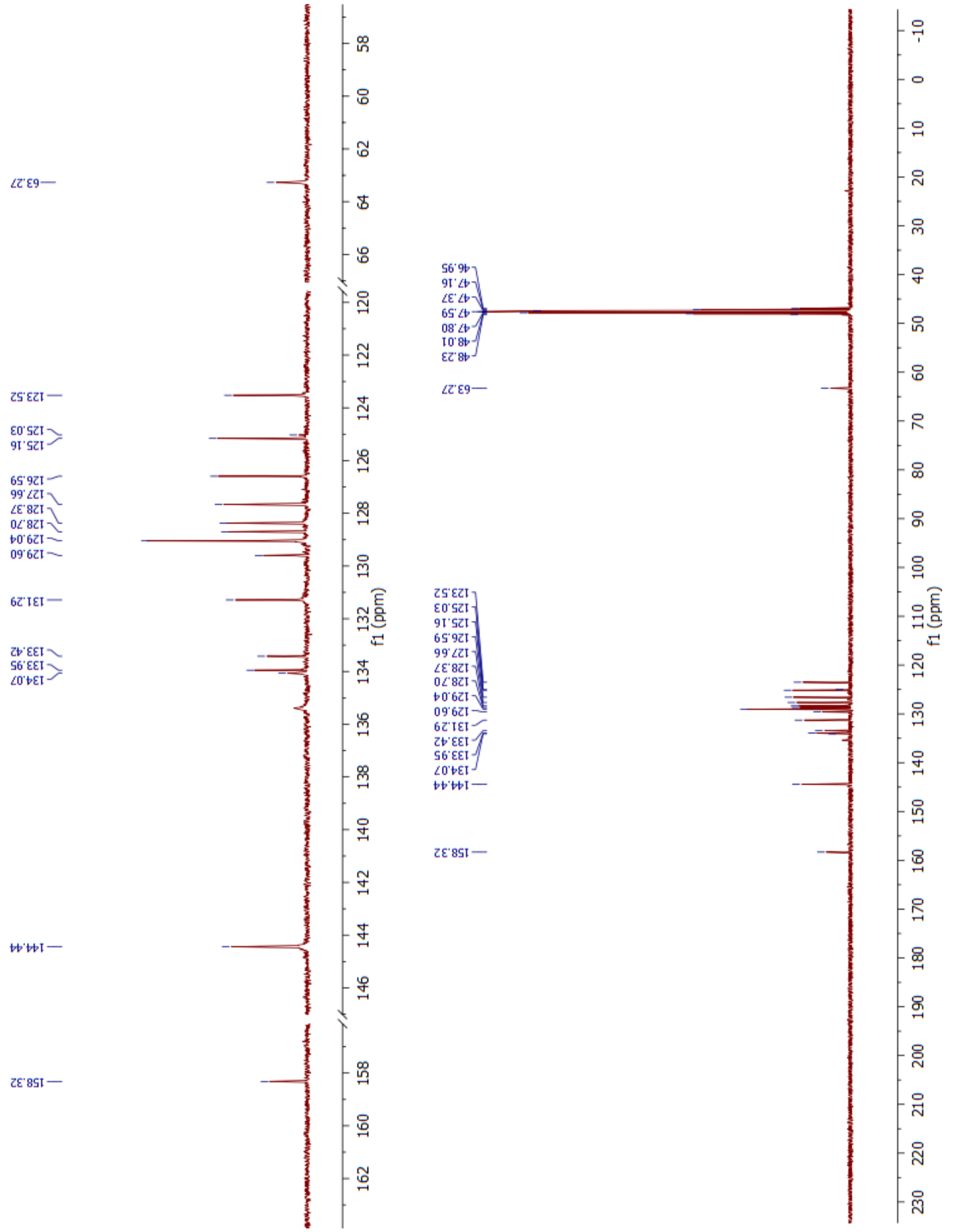

Figure S-10. ^{13}C-NMR spectra (**1**)

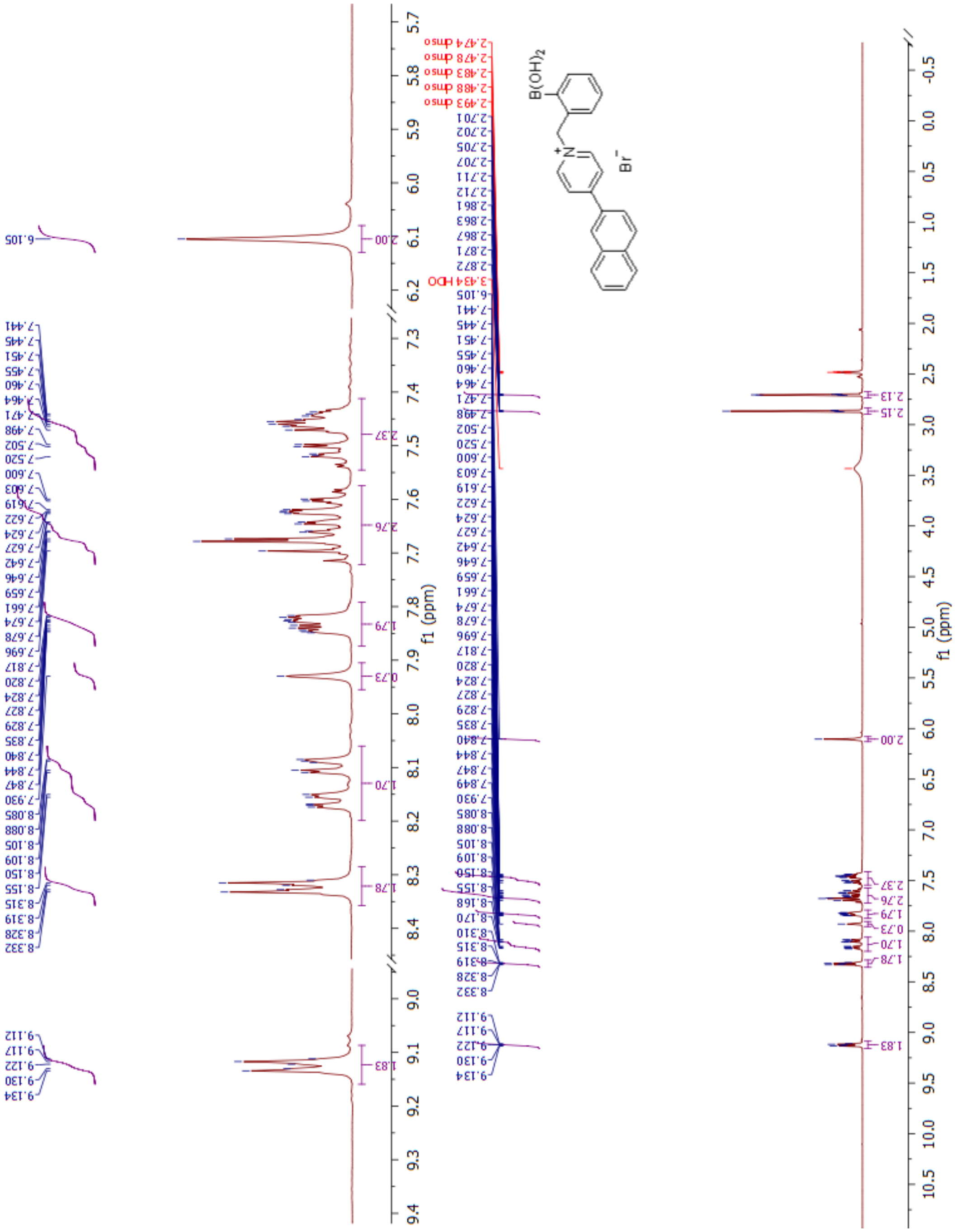

Figure S-11. ^{1}H-NMR spectra of (**2**)

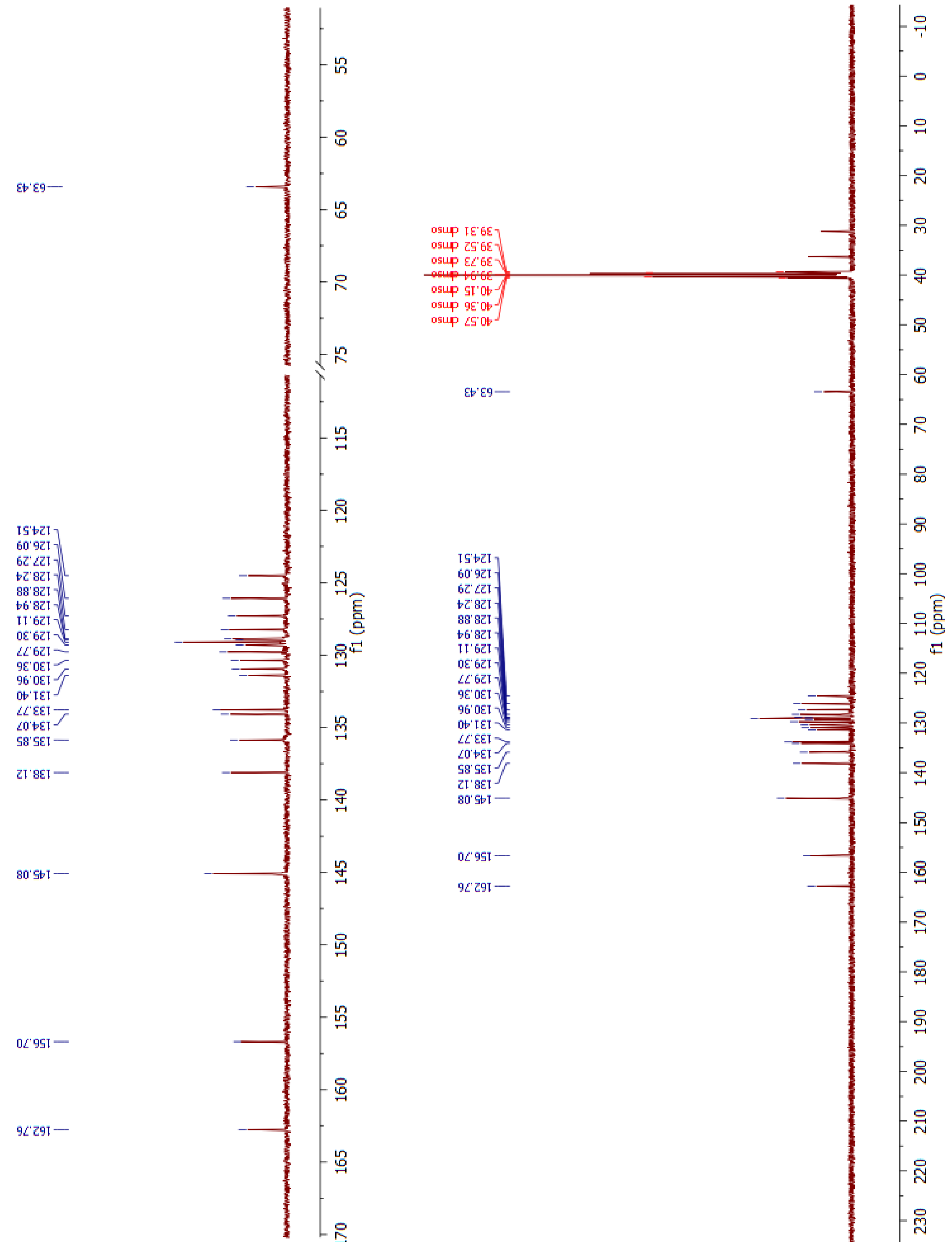

Figure S-12. ^{13}C-NMR spectra of (**2**)

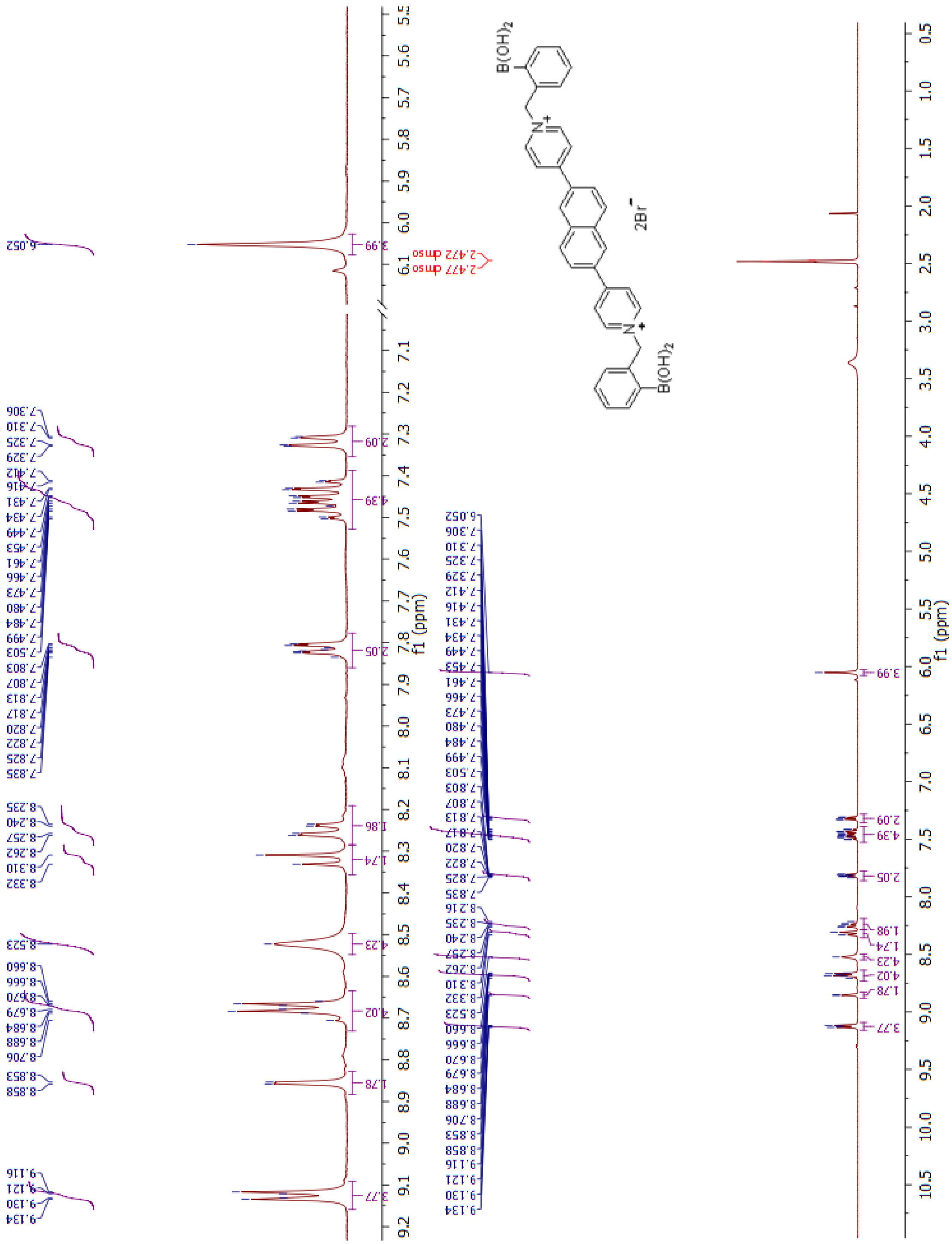

Figure S-13. ^{1}H-NMR spectra of (**3**)

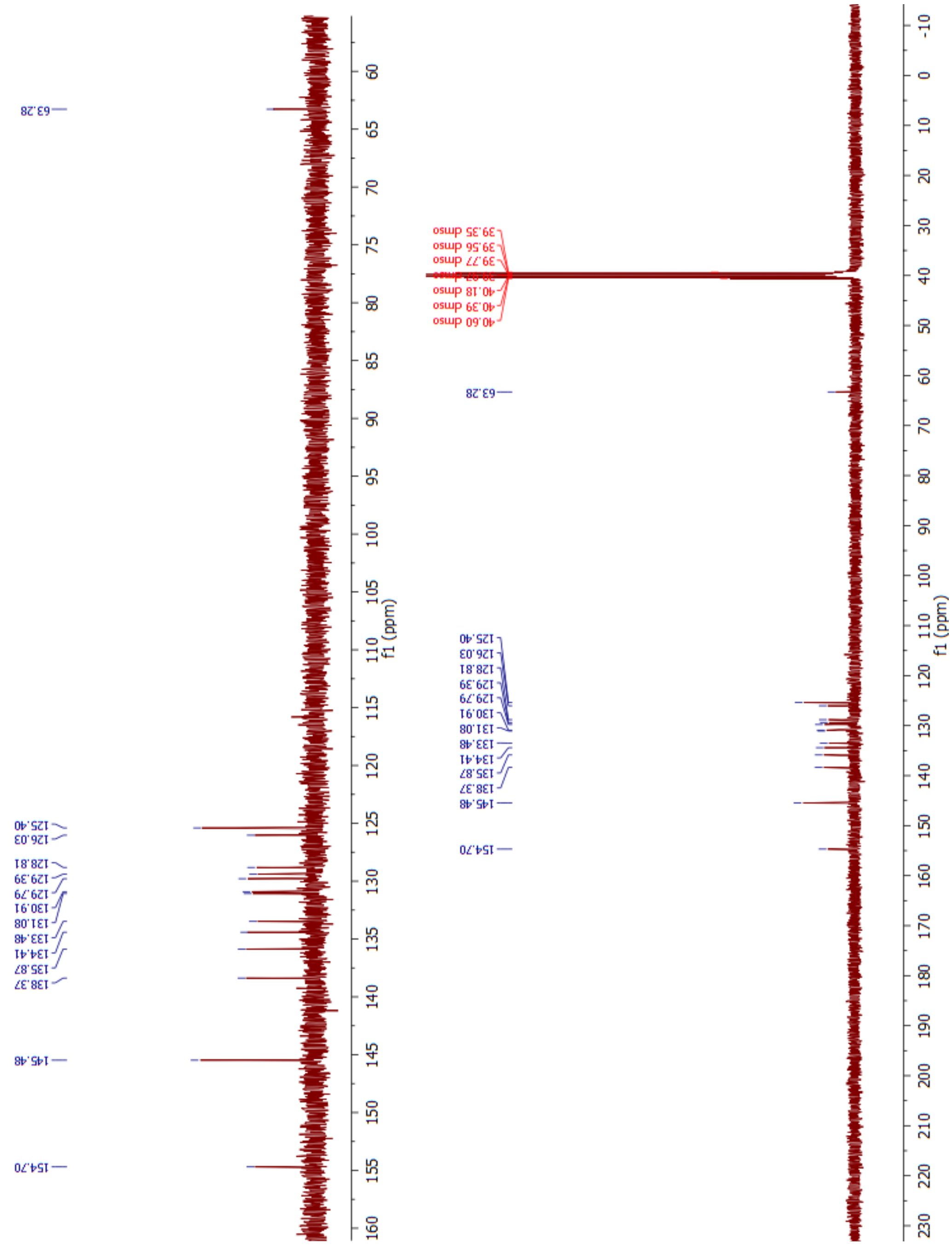

Figure S-14. ^{13}C-NMR spectra of (**3**)

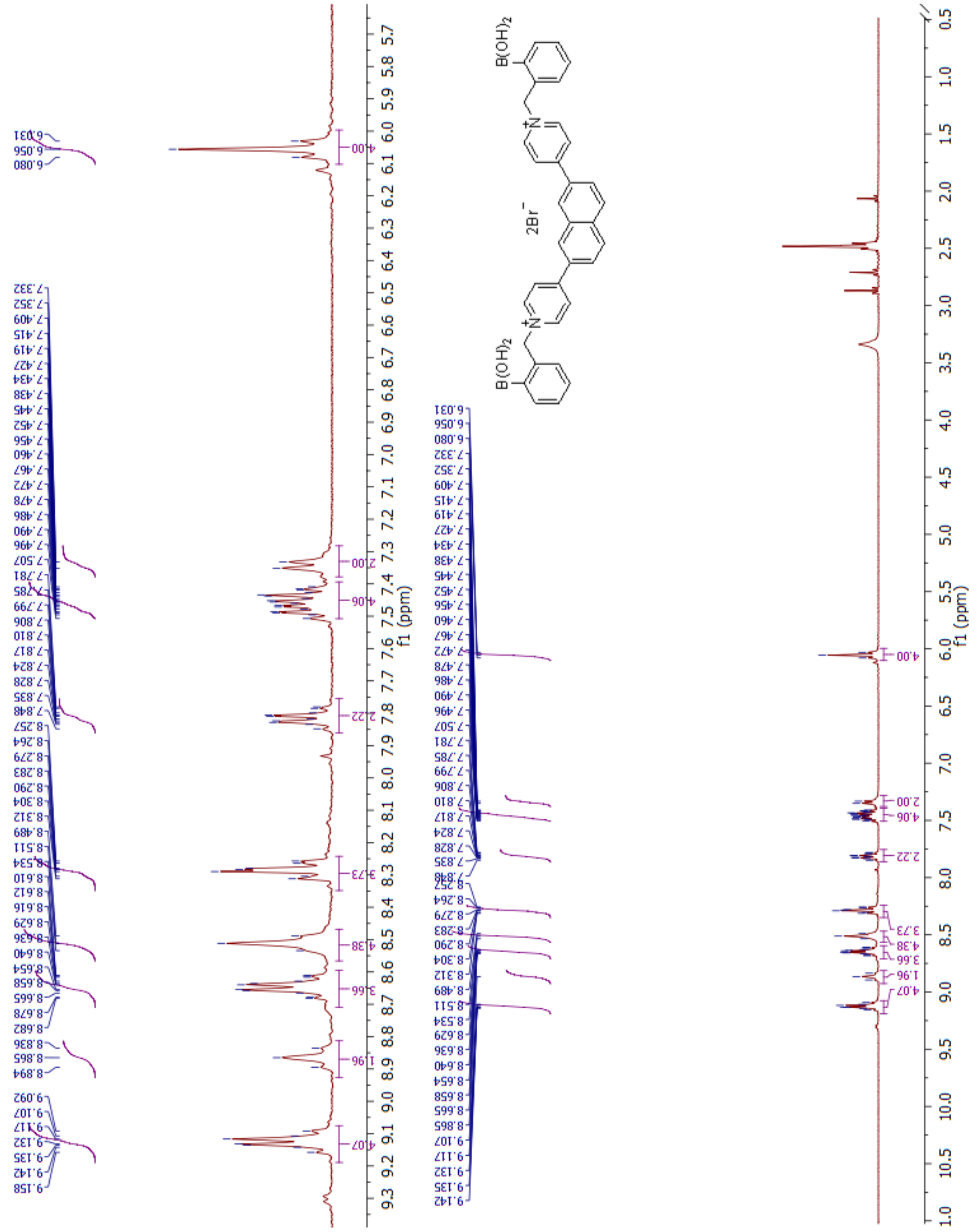

Figure S-15.^{1}H-NMR spectra of (**4**)

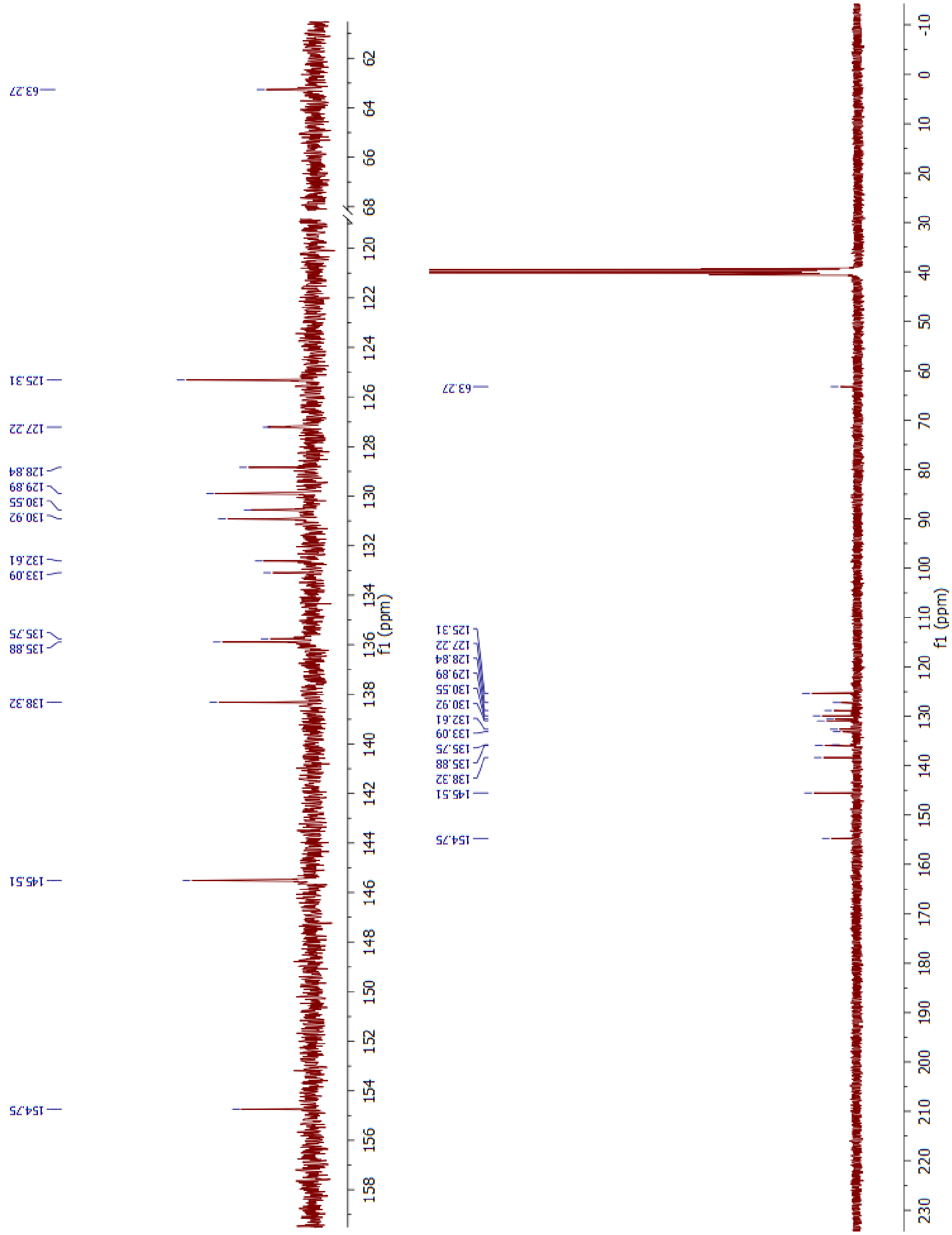

Figure S-16. ^{13}C-NMR spectra (**4**)

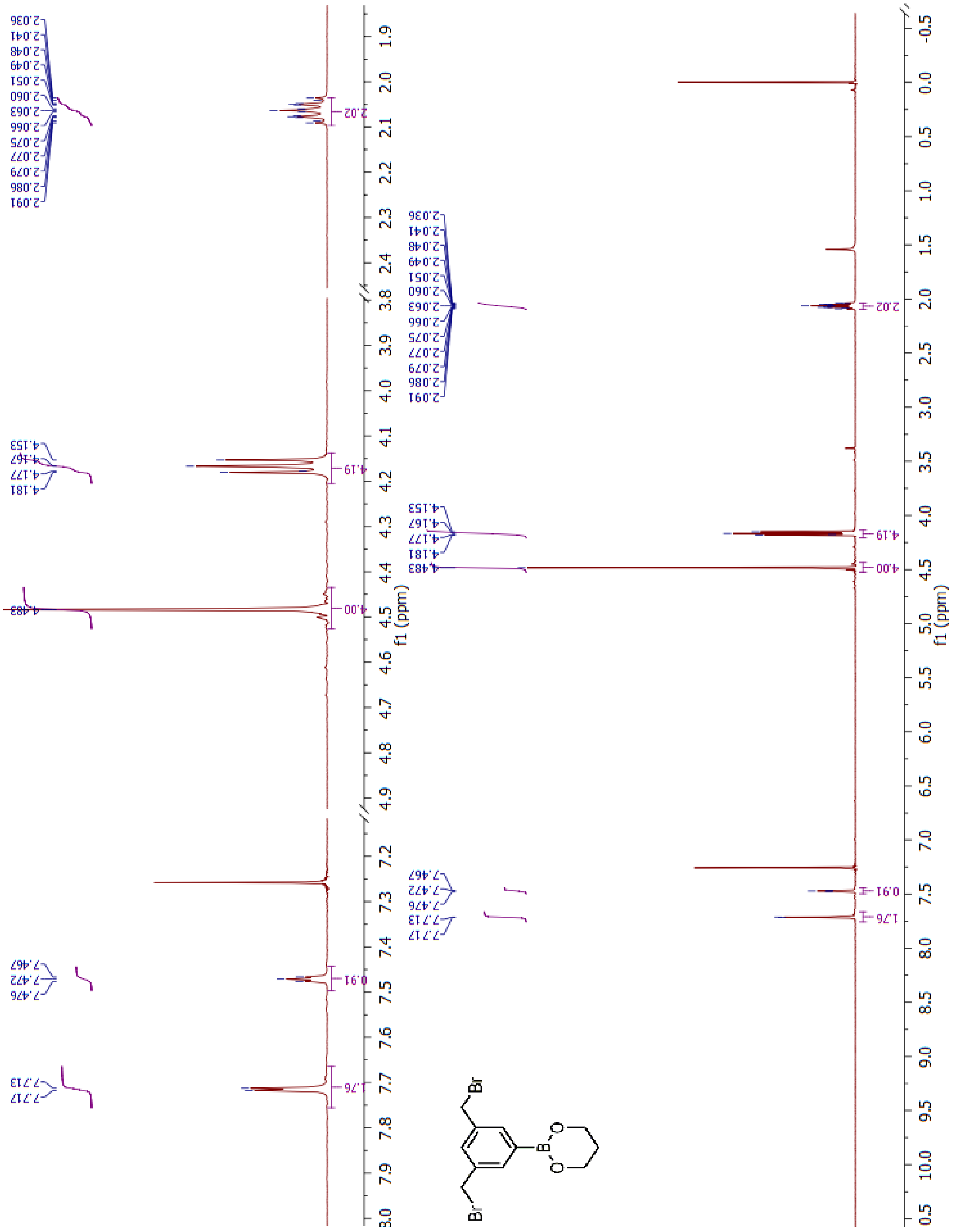

Figure S-17.^{1}H-NMR spectra of (**borinane**).

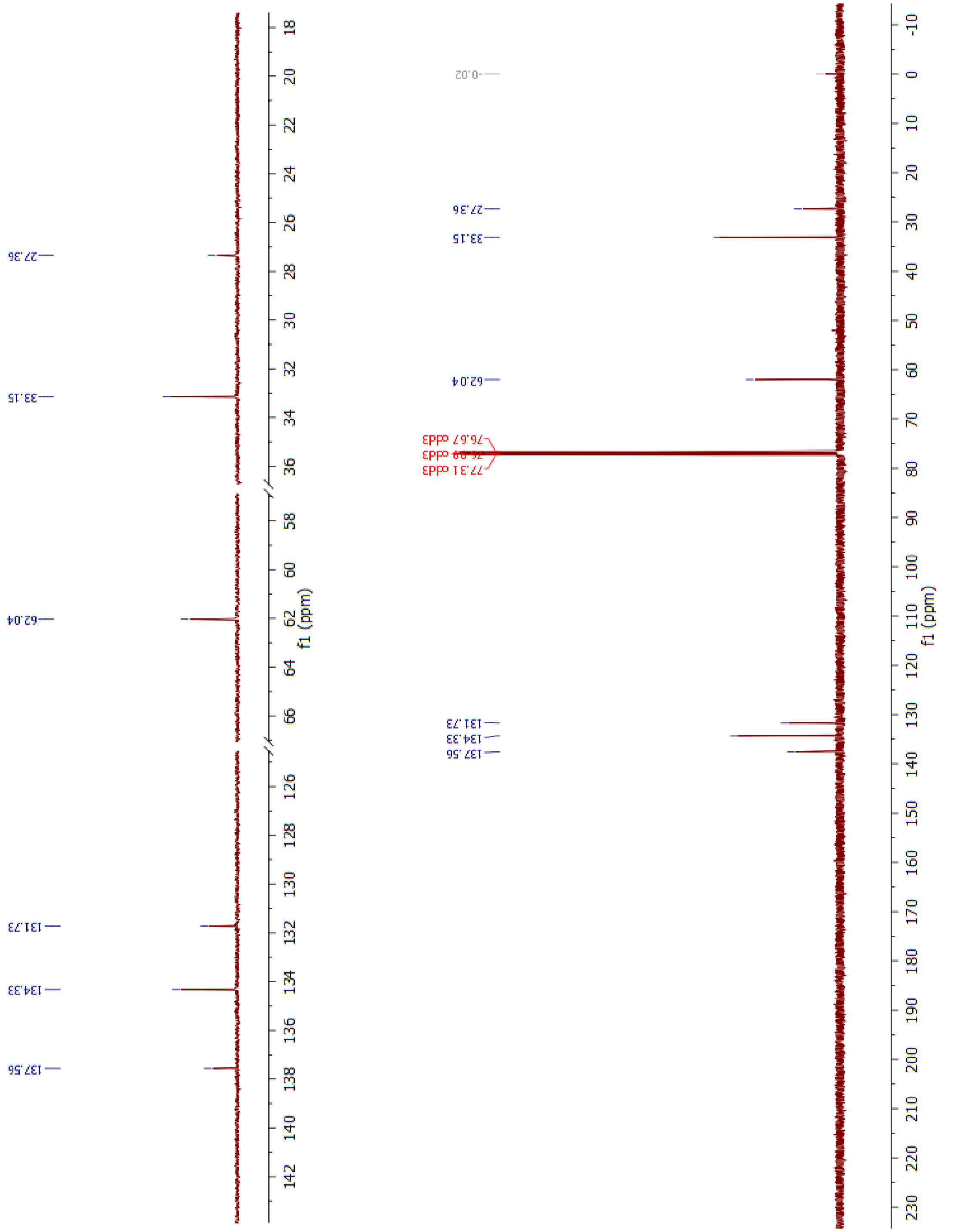

Figure S-18. ^{13}C-NMR spectra of (**borinane**)

4. References

1 Suri, J. T., Cordes, D. B., Cappuccio, F. E., Wessling, R. A. & Singaram, B. Continuous glucose sensing with a fluorescent thin-film hydrogel. *Angew Chem-Intl Ed* **42**, 5857-5859 (2003).

2 Karagiaridi, O. *et al.* Opening Metal-Organic Frameworks Vol. 2: Inserting Longer Pillars into Pillared-Paddlewheel Structures through Solvent-Assisted Linker Exchange. *Chem Mat* **25**, 3499-3503, (2013).

3 Yang, J., Liu, S., Zheng, J.-F. & Zhou, J. Room-Temperature Suzuki-Miyaura Coupling of Heteroaryl Chlorides and Tosylates. *Eur J Org Chem*, 6248-6259, (2012).
